# Selenium‐Based Nanoplatforms: An Emerging Theranostic Paradigm for Gynecological Cancers

**DOI:** 10.1002/advs.75245

**Published:** 2026-04-23

**Authors:** Hejing Liu, Yujia Zhou, Zihan Zhang, Rong Ma, Luyi Ruan, Liqing Miao, Boxin Zhang, Huihui Ji, Tianfeng Chen, Xueqiong Zhu

**Affiliations:** ^1^ Zhejiang Provincial Clinical Research Center for Gynecological Diseases Department of Obstetrics and Gynecology The Second Affiliated Hospital of Wenzhou Medical University Wenzhou Zhejiang P. R. China; ^2^ Department of Chemistry State Key Laboratory of Bioactive Molecules and Druggability Assessment MOE Key Laboratory of Tumor Molecular Biology Jinan University Guangzhou P. R. China

**Keywords:** diagnosis, gynecological cancers, selenium, selenium‐based nanoparticles, therapeutic

## Abstract

Gynecological cancers present significant therapeutic challenges due to heterogeneity, drug resistance, and the lack of precise diagnostic tools. Selenium (Se), an essential trace element with intrinsic anticancer activity, has emerged as a promising candidate. Numerous epidemiological studies have confirmed a significant inverse correlation between selenium level and the risk of gynecological cancers’ development, and a low selenium status portends a poorer prognosis. Of note, the value of selenium extends beyond as the potential biomarker that selenium supplementation acts as both a chemopreventive agent and a therapeutic adjuvant in oncology. However, the current understanding of the mechanism of various forms of selenium in the treatment of gynecological cancers remains insufficient. Therefore, this review firstly summarizes the advances of various selenium species (inorganic, organic, and selenium‐based nanoparticles) for the treatment of gynecological cancers. Among these, selenium‐based nanoparticles have become a potential candidate for the treatment of gynecological cancers due to their higher bioavailability, better anticancer activity, and lower toxicity. This review systematically highlights their multifaceted therapeutic mechanisms and the applications in cancer diagnosis and imaging. As the platforms that converge diverse treatment modalities with diagnostic functions, Se‐based nanoparticles provide new insights into the therapeutic and early diagnosis applications in gynecological cancers.

## Graphical abstract

This reivew summarizes Selenium as a multifunctional anticancer regulator in gynecological cancers. It reduces tumor risk, enhances therapeutic efficacy, and reduces treatment toxicity. Selenium also overcomes chemoraditherapy resistance, improving overall treatment outcomes.

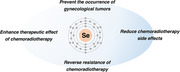

**Abbreviations**
AACES: African American Cancer Epidemiology Study
ABC transporters: ATP‐binding cassette transporters
ALT: alanine aminotransferase
AST: aspartate aminotransferase
Au: aurum
BRCA1: breast cancer susceptibility gene 1‌
CA125: cancer antigen 125
Cd‐Se: cadmium selenide
CDT: chemodynamic therapy
CHO: cholesterol
Cht: chitosan
CIK: cytokine‐induced killer cells
CMP: carboxymethyl cottonin
CREA: creatinine
CRT: chimeric antigen receptor T‐cell
CTLs: cytotoxic T lymphocytes
Cu: copper
Cys: cystine
CySH: cysteine
DCs: dendritic cells
dGMPSe: 2'‐deoxyguanosine‐5'‐O‐selenophosphate
DMSe: dimethyl selenide
DOX: doxorubicin
DSA: Diselenide 9, 10‐styrene‐Artemisia
EBRT: external beam radiation
EPR: enhanced permeability and retention effect
FA: folate
FAR: folate receptor
Fe: iron
FFQ: food frequency questionnaire
FIGO: Federation International of Gynecology and Obstetrics
GLU: glucose
GPxs: glutathione peroxidases
GSH: glutathione
GSSeH: selenenylsulfide
GSSeSG: selenodiglutathione
HA: hyaluronic acid
HE4: human epididymis protein 4
HPLC‐ICP‐MS: high performance liquid chromatography‐inductively coupled plasma mass spectrometer
HPV: human papillomavirus
HSA: human serum albumin
ICD: immunogenic cell death
IMRT: intensity modulated radiation therapy
LNT/ LET: lentinan
mAb: monoclonal antibody
MBs: microbubbles
MeSeA: methylseleninic acids
MetSeCys: methylselenocysteine
MPA: mercaptopropionic acid
MRI: magnetic resonance imaging
NCs: nanocomposites
NDs: nanometer dots
NIR: near‐infrared
NK cells: natural kill cells
NPs: nanoparticles
OH: hydroxyl radicals
Pd: palladium
PDA: polydopamine
PEG: polyethylene glycol
PTT: photothermal therapy
PTX: paclitaxel
QDs: quantum dots
ROS: reactive oxygen species
RT: radiotherapy
Ru: ruthenium
SA: sialic acid
SBRT: stereotactic body radiation therapy
Se: selenium
SeCys: selenocysteine
SeCys_2_
: selenocystine
SeD: selenodiazole derivative
SeMet: selenomethionine
SeNPs: selenium nanoparticles
TMEs: tumor microenvironments
TrxRs: thioredoxin reductases
UA: uric acid
UREA: urea
VEGF: vascular endothelial growth factor
WHO: World Health Organization
XR: X‐ray resistance

## 1 Introduction

Gynecological cancers encompass cervical, endometrial, ovarian, vulvar, vaginal, and fallopian tube cancers. Cervical, endometrial, and ovarian cancers are the most frequently diagnosed malignancies, presenting a major risk to women's lives and health. Cervical cancer ranks as the fourth most frequently diagnosed malignancy in women worldwide [1]. Its occurrence is primarily attributable to human papillomavirus (HPV) infection [2]. While the World Health Organization (WHO) has proposed eliminating cervical cancer by 2030, most low‐ and middle‐income countries are still far from achieving this goal because of differences in medical standards [3]. High‐income countries have the highest incidence of endometrial cancer, and its prevalence is on the rise globally [4]. Ovarian cancer ranks fifth in terms of cancer‐related mortality among all gynecologic malignancies, which is considered the most lethal gynecologic malignancy. Due to the absence of reliable screening modalities and distinct symptoms, diagnosis is often delayed, typically at an advanced stage, and recurrence follows initial treatment at a high rate [5, 6]. Currently, the major therapies for primary gynecological cancers are still surgery, radiotherapy, and chemotherapy. Radiotherapy (RT) serves as a cornerstone of treatment strategies for select individuals diagnosed with cervical or endometrial cancers [7, 8, 9, 10]. The spectrum of clinical radiation modalities encompasses external beam radiotherapy (EBRT), brachytherapy, and the application of radioisotopes [11, 12]. For example, in gynecologic oncology, EBRT now incorporates advanced techniques such as intensity‐modulated radiation therapy (IMRT) [13], stereotactic body radiation therapy (SBRT) [14], and proton therapy [15]. Modern technological advancements have improved precision and control, enhanced treatment effectiveness while reducing side effects [11]. Different from other cancers, the treatment of gynecologic cancers presents several particularly prominent issues. (1) During radiotherapy for pelvic malignancies, the healthy bowel and bladder invariably lie within the radiation field, leading to conditions such as radiation enteritis and cystitis for which there are few effective treatment options [16, 17]. Therefore, exploring new strategies to enhance the efficacy and reduce the toxicity of radiotherapy has great clinical importance. (2) Due to the limited drug selectivity in gynecological tumors, platinum‐based drugs remain the first‐line chemotherapy. Although the initial response to platinum therapy is favorable, most patients eventually develop resistance through multiple biological mechanisms. resulting in treatment failure [18]. Similarly, the radioresistance of cancer remains a critical limitation of radiotherapy, often leading to increased risks of locoregional relapse and distant metastasis. [19]. (3) For women undergoing cancer treatment, ovarian damage from chemotherapy or radiotherapy is a particularly serious side effect, which can disrupt ovarian hormone levels, reduce or lose fertility, and elevate the risk of premature menopause [20]. Providing the best option for fertility preservation for gynecological cancer patients is also an important proposition for gynecologists. Therefore, New diagnostic and therapeutic methods need to be further developed for the prevention and treatment of gynecological tumors.

As a naturally occurring trace element, Selenium (Se) is crucial for various physiological processes in humans [21]. In contrast to other trace elements, the Se element is directly integrated into proteins as the 21st amino acid, known as selenocysteine (SeCys). There are 25 selenoproteins in humans, and changes in the Se status or expression of selenoproteins can affect nearly every organ in the body [22]. The high risk of breast cancer [23], thyroid cancer [24], and prostate cancer [25] has been linked to Se deficiency. Abundant epidemiological evidence has revealed an inverse correlation between Se and the risk of gynecological cancers. Clinical data further demonstrate that Se level in patients with gynecological cancers is significantly lower than that in healthy controls [26, 27, 28, 29]. Moreover, Se deficiency is frequently associated with advanced clinical stage and poorer prognosis, indicating that low Se status may serve as a potential indicator of both cancer risk and disease severity in gynecological cancers [29, 30, 31]. Importantly, supplementation with 20 µg of Se per day has been shown to reduce the incidence of ovarian cancer by approximately 30% [32], and women receiving Se supplementation display a two‐fold lower risk of developing breast cancer susceptibility gene 1 (BRCA1) ‐associated ovarian cancer compared with those not taking Se supplements [33], suggesting a protective role of Se in preventive interventions for genetically susceptible populations. These data reveal that Se is not only a promising biomarker for the early detection and prognostic evaluation of gynecological cancers, but also sparks academic inquiry into its role in therapeutic intervention and prognostic management.

Of note, the role of Se is gradually shifting from a chemopreventive drug to a therapeutic adjuvant. In the treatment strategies for gynecological cancers, Se has shown unique and multidimensional anti‐tumor potential. Due to its triple efficacy of synergistic enhancement, side effects reduction, and reversal of drug resistance, Se is emerging as a highly promising therapeutic strategy [34]. More precisely, by promoting the synthesis of antioxidant enzymes, specifically thioredoxin reductases (TrxRs) and glutathione peroxidases (GPxs), Se at nutritional (low) concentrations maintains intracellular redox homeostasis. Under this physiological level, characterized by the saturation of selenoproteins, effectively shields healthy cells from oxidative damage triggered by reactive oxygen species (ROS) [21, 35], which can be used to reduce the toxicity of radiotherapy and chemotherapy in gynecological cancers [36]. Notably, Se plays an essential role in ovarian follicle growth and maturation, primarily through its antioxidant effects during follicular development [37, 38, 39]. At pharmacological concentrations (high concentrations), it acts as a pro‐oxidant, causing cancer cells to undergo oxidative stress due to the strong generation of ROS through the redox cycle [21]. The dosage causing the prooxidant and toxic effects induced by Se compounds varies in different tissues or cells. In addition to its antiviral effect achieved by modulating the function of both adaptive and innate immune cells [40], Se supplementation is also associated with anti‐tumor immunostimulating effects, including an increase in cytotoxic lymphocyte‐mediated cancer cytotoxicity, enhancing T cells’ proliferation, and activating natural killer cells’ function [41]. Therefore, Se is suitable for treatment regimens that concurrently target both HPV virus infection and cervical cancer. Conventional Se includes both inorganic and organic Se compounds. Among the inorganic compounds found in various foods, Se and selenite are the most important. Methylselenocysteine (MetSeCys), selenomethionine (SeMet), and SeCys are the most common organic compounds [21, 42], which vary in chemical structure and show different pharmacological activity. Additionally, many new inorganic or organic Se compounds have been designed and developed for the treatment of various tumors [34]. At present, several Se compounds have been approved for clinical Se supplemental treatment, such as Se yeast capsules [43] and Se solution called Sel Vita Gen [33]. Both organic and inorganic Se compounds have been studied as promising candidates for clinical gynecological cancers’ therapy, either in combination with other treatments [21, 42].

Nanomedicine is an emerging therapeutic approach designed to improve drug delivery and treatment effectiveness while minimizing or eliminating adverse side effects on normal tissues, and it has gradually been promoted and applied to the diagnosis and treatment of cancers [44, 45]. Se nanoparticles exhibit higher absorption rate, enhanced biological activity, and greater bioavailability compared to elemental Se, which are currently widely studied as an antioxidant, antibacterial, and anticancer agent [46]. Functionalized Se‐based nanoparticles are emerging as a major therapeutic platform, especially in anticancer therapy, either as standalone treatments or synergistic therapies with radiotherapy and chemotherapy, tumor immunotherapy, and other cancer therapies [47]. According to the characteristics of cancers and the tumor microenvironment with a weak acidity and hypoxia state, Se‐based nanoparticles can actively and passively target tumor sites and respond to release active components. Consequently, tumor cells are killed effectively while normal tissues are spared from toxic effects and adverse effects [48]. Additionally, in tumor diagnosis, the application of Cd‐Se quantum dots (QDs) in laser irradiation, imaging, and biomedical usage further enhances the use of Se as a semiconductor material. Its unique photoluminescent property allows for more accurate diagnosis of gynecological cancers at earlier stages [49].

**FIGURE 1:**
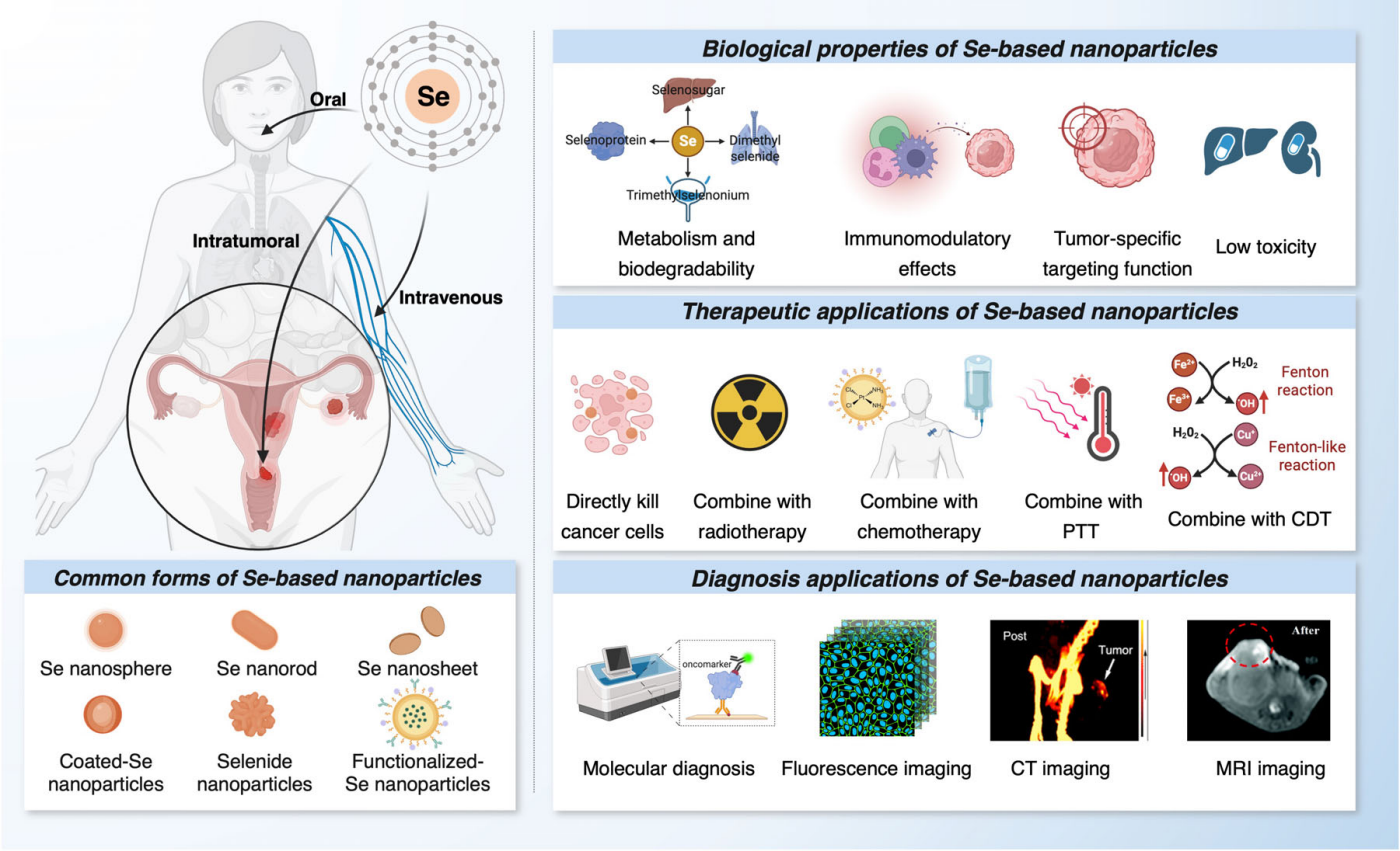
Biomedical applications of Se‐based nanoparticles in gynecological cancers: schematic overview. This schematic illustration presents the common structural forms of Se‐based nanomaterials and the administration routes, including oral, intravenous, and intratumoral delivery, and also summarizes the biological properties, therapeutic applications, and diagnostic applications of Se‐based nanoparticles. The images in the lower right were reproduced with permission [143]. Copyright 2015, American Chemical Society and [145], Copyright 2022, Royal Society. Others were created with BioRender.com.

Therefore, Se compounds and Se‐based nanoparticles closely align with the needs of clinical treatment for gynecological tumors. In this review, based on epidemiological surveys, the relationship between Se intake, blood Se content, and the risk of gynecological cancers is summarized. Se supplement has a synergistic effect of enhancing efficacy and reducing side effects when used in combination with radiotherapy or chemotherapy. It then discusses the anti‐tumor effects of Se compounds in various forms. To better understand the efficacy of nanoparticles, their physicochemical and biological properties are also reviewed. In addition, the anti‐tumor effects of Se‐based nanoparticles in gynecological cancers, including direct killing, radiotherapy sensitization, chemotherapy drug delivery, chemotherapeutic sensitization, combination with photothermal therapy (PTT), and combination with chemodynamic therapy (CDT), are compiled. Finally, the applications of Se‐based nanoparticles as probes and contrast agents in cancer diagnosis and imaging are concluded. Overall, Se and Se‐based nanoparticles have extensive potential in the field of female reproductive cancer therapy and diagnosis (Figure 1).

## 2 Clinical Studies of Se in Gynecological Cancers

**FIGURE 2:**
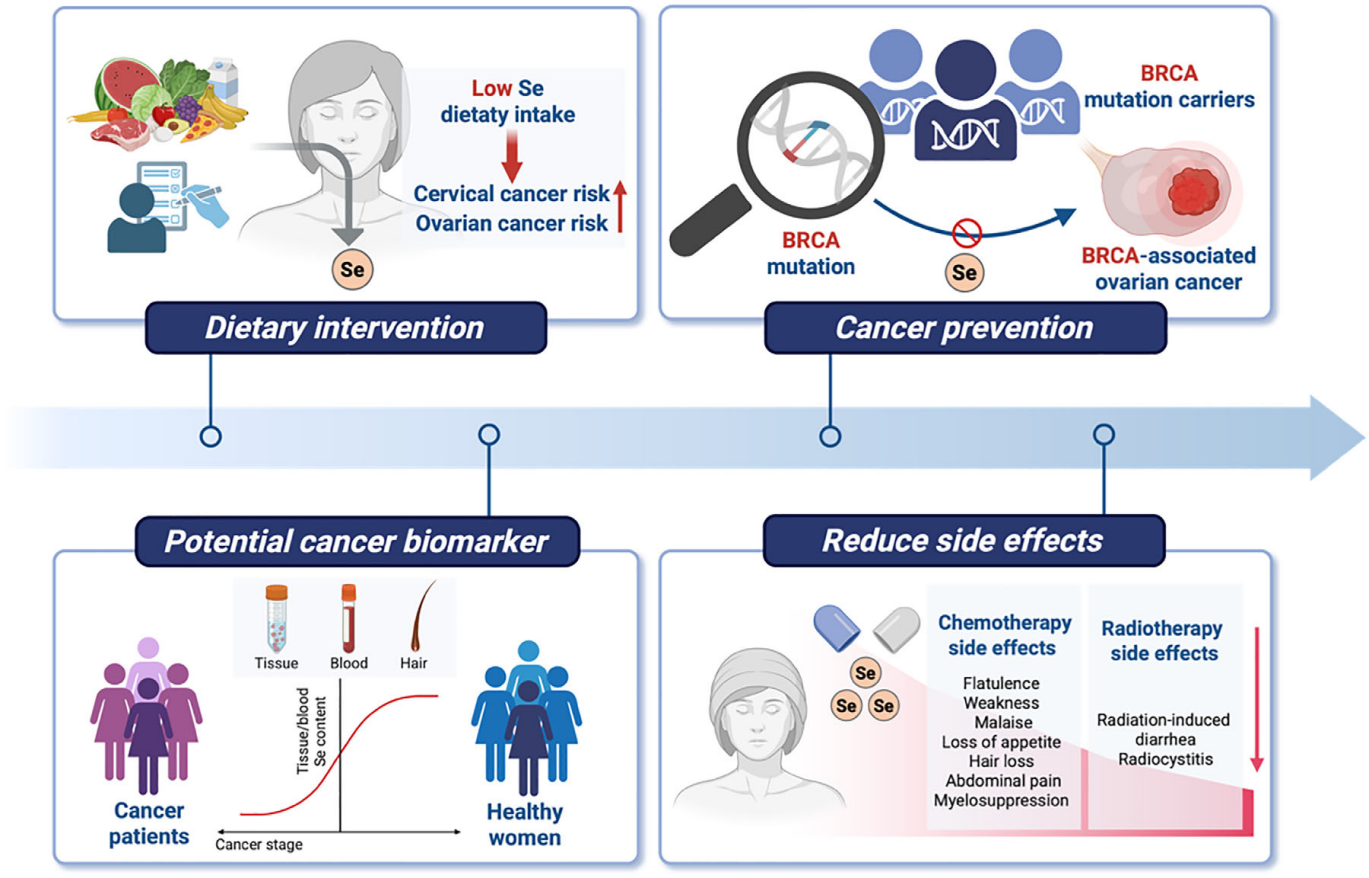
Epidemiological evidence and clinical research of Se in gynecological cancers. (Top left) Dietary intervention to address Se deficiency‐induced cancer risk; (Bottom left) Se as the potential biomarkers in cancer diagnosis; (Top right) Cancer prevention of Se specifically for BRCA1‐associated ovarian cancer; (Bottom right) Se alleviate side effects induced by chemotherapy and radiotherapy (Created with BioRender.com).

This section provides multi‐level evidence for understanding the role of Se in the prevention and treatment of gynecological cancers, and explores the clinical potential of its use in nutritional intervention or adjuvant therapy (Figure 2 and Table 1).

**TABLE 1:** Summary of clinical research on Se and gynecological cancers.

Classification	Researcher and reference	Cancer types	Research object	Key findings
Dietary intervention	Nazari et al. [50]	cervical cancer	2088 females	Higher dietary Se intake is linked to a reduced risk of cancer and slower disease progression.
	Zhu et al. [51]	endometrial cancer	12,437 females	No statistically significant relationship found between dietary Se consumption and cancer risk.
	Terry et al. [32]	ovarian cancer	406 cancer cases and 632 controls	Supplemental Se intake (>20 µg/d) is associated with a 30% lower risk of ovarian cancer.
	Gifkins et al. [52]	ovarian cancer	205 cancer cases and 390 healthy volunteers	Food‐source Se consumption is negatively correlated with ovarian cancer risk.
Blood Se concentration	Thompson et al. [53]	cervical cancer	227 cancer cases and 526 controls	No correlation observed between serum Se concentration and the occurrence of invasive cancer.
	Cunzhi et al. [27]	cervical cancer	40 cancer cases and 50 healthy subjects	Patients with cervical cancer exhibited lower serum Se level compared to healthy individuals.
	Okunade et al. [54]	cervical cancer	50 cancer cases without treatment and 100 volunteers	Cervical cancer patients had significantly lower serum Se level than control volunteers
	Kim et al. [30]	cervical cancer	Total 136 patients	Plasma Se level decrease as tumor stages progress from benign to advanced/recurrent.
	Qi et al. [31]	cervical cancer	29 cancer cases with poor prognoses and 29 cancer cases with better prognoses	Lower plasma Se level is associated with poor prognosis compared to better prognosis.
	Janowska et al. [28]	endometrial cancer	153 cancer cases and 153 healthy women	Average serum Se concentration is lower in patients than in healthy controls.
	Kluza et al. [29]	ovarian cancer	157 cancer cases and 157 healthy volunteers	Lower serum Se in patients; levels further decrease in advanced FIGO stages.
	Caglayan et al. [55]	ovarian cancer	26 patients and 46 healthy volunteers	Patients with malignant epithelial ovarian cancer exhibit reduced erythrocyte Se levels.
Tissue & Hair Se level	Cunzhi et al. [27]	cervical cancer	40 cancer cases and 50 healthy subjects	Se content in cancer tissues is significantly lower than in paired non‐lesion tissues.
	Wadhwa et al. [56]	cervical cancer	31 cancer cases	Se level in scalp hair is significantly lower (51%‐53% lower for ovarian) in patients.
	Canaz et al. [57]	ovarian cancer	20 malignant epithelial ovarian cancers, 15 epithelial borderline tumors, and 20 non‐neoplastic healthy ovaries	No statistical difference found in Se concentration between malignant, borderline, and healthy ovaries.
Cancer prevention	Kowalska et al. [58]	BRCA1 mutation carriers	26 BRCA1 mutation carriers and 26 case‐control pairs	Heterozygous carriers with deleterious BRCA1 gene mutations exhibited a decreased frequency of chromosome breaks
	Huzarski et al. [33]	BRCA1 mutation carriers	200 healthy BRCA1 mutation carriers and 100 case‐control pairs	Se supplementation leads to a two‐fold lower risk of BRCA1‐associated ovarian cancer.
Reduce side effects	Yang et al. [59]	cervical cancer	104 patients diagnosed with FIGO stage IIB cervical cancer	Se yeast (200 µg/daily) dramatically reduces grade 3 myelosuppression and thrombocytopenia during chemoradiotherapy.
	Muecke et al. [60]	cervical cancer and uterine cancer	11 cancer cases and 70 uterine cancer cases	Sodium selenite supplement orally significantly decreases the incidence and severity of radiation‐induced diarrhea.
	Sieja et al. [61]	ovarian cancer	62 cancer cases	Se supplementation (200 µg/daily) reduces chemotherapy side effects like flatulence, hair loss, weakness, and malaise.

### 2.1 Dietary Intake of Se and the Occurrence of Gynecological Cancers

As an essential trace element, Se is primarily found in food, but its content varies based on the geographical location, soil quality, and plant accumulation [62]. Recently, many studies, both on animal experiments and clinical studies, have shown that there is a correlation between the lower Se dietary intake and a higher cancer risk [23, 63]. Using the questionnaire, researchers studied the relationship between dietary Se intake and the occurrence of gynecological cancers. Typical questionnaire including the Food Frequency Questionnaire (FFQ).

According to Nazari et al. [50], dietary intake of Se was linked to a reduced risk of cervical cancer and its progression by investigating the essential micronutrient Se in 2088 healthy subjects and patients with cervical cancer using a semi‐quantitative FFQ.

In another study, a total of 12 437 female participants aged over 20 were recruited from the American National Health and Nutrition Examination Survey database to examine the relationship between dietary Se consumption and the incidence of endometrial cancer. No statistically significant relationship was observed between dietary Se consumption and endometrial cancer risk [51].

In their research on ovarian cancer, Terry et al. [32] assessed the link between dietary Se intake and the risk of ovarian cancer among African‐American women using data from the African American Cancer Epidemiology Study (AACES), which was based on FFQ, containing 406 ovarian cancer cases and 632 controls, using multivariable logistic regression models. Results showed that there was a 30% lower risk of ovarian cancer among women with supplemental Se intakes (>20 µg/d) compared to those without. In a case‐control study using the FFQ, Gifkins et al. [52] examined the connection between individual Se intake and the risk of ovarian cancer. This study enrolled 205 cases of ovarian cancer and 390 healthy volunteers, in which food‐source Se consumption was found to be negatively related to ovarian cancer risk.

The heterogeneous effects of dietary Se intake across different gynecological cancers may be attributed to differences in tumor etiology. Meanwhile, variations in estrogen and progesterone levels, as well as regional differences in Se distribution, may also contribute to biases in the observed findings. However, there is currently no direct evidence to support these explanations. Future studies are needed to clarify the association between dietary Se intake and gynecological malignancies. Overall, Se intake is negatively correlated with the risk of cervical and ovarian cancer, suggesting its potential as a tumor preventive agent.

### 2.2 Blood Se Concentration and the Occurrence, Development, and Prognosis of Gynecological Cancers

The role of microelements in blood with cancer oncogenesis has been the subject of intense research. In this section, the relationship between blood Se content and the occurrence of gynecological cancers is summarized.

Thompson et al. [53] determined the serum Se content in 227 invasive cervical cancer cases and 526 controls, which were selected by random‐digit dialing from the same communities, to determine if serum Se was associated with cervical cancer. However, results showed that there was no correlation between serum Se concentration and the occurrence of invasive cervical cancer. To examine the association between the content of Se in serum and cervical cancer, He et al. [26] conducted systematic reviews and meta‐analyses. The findings indicated that individuals with cervical cancer had significantly lower serum Se content compared to healthy females or women with benign uterine diseases. Similarly, Cunzhi et al. [27] measured serum Se content in 40 cases of patients with cervical cancer compared with 50 healthy subjects. Patients with cervical cancer exhibited lower serum Se level compared to healthy individuals. Okunade et al. [54] enrolled 50 patients diagnosed histologically with cervical squamous cell cancer who had not received any treatment, as well as 100 volunteers free of cancer. As expected, cervical cancer patients had significantly lower serum Se level than control volunteers. Based on the stage of the cancer, Kim et al. [30] divided 136 patients into five groups. Group 1 exhibited uterine benign tumors, which consisted of women with adenomyosis, myoma, mild cervical dysplasia, benign ovarian cyst, and menopausal state. In groups 2–5, malignant tumors were diagnosed with four subgroups, including preinvasive cervical lesions, cervical cancer in early stages, cervical cancer in advanced stages, and recurrent cervical cancer. Compared to benign and pre‐invasive cervical cancer patients, advanced‐stage and recurrent cancer patients had significantly lower serum Se concentration. As tumor stages progressed from benign to malignant and recurrence, plasma Se level decreased proportionally. Qi et al. [31] conducted a study to examine the relationship between blood Se level and the prognosis of cervical cancer patients. This study included 29 cervical cancer cases with poor prognoses and 29 with better prognoses. Plasma Se level, as determined by an atomic fluorescence spectrometer, was lower in the group with poor prognosis compared to the group with better prognosis.

Janowska et al. [28] utilized a mass spectrometer with excitation in inductively coupled plasma to assess the Se content in serum samples from 153 endometrial cancer patients and 153 healthy women. They found that the average Se concentration was lower in patients with endometrial cancer compared to the healthy controls.

Kluza et al. [29] enrolled 157 ovarian cancer patients and 157 healthy volunteers, discovering that the diseased group had a lower average serum Se concentration compared to the control group. Moreover, Se content was found to decrease in ovarian cancer patients with an advanced stage according to the Federation International of Gynecology and Obstetrics (FIGO) classification. Caglayan et al. [55] conducted a study involving 26 patients diagnosed with epithelial ovarian cancer and 46 healthy volunteers. They found that individuals with malignant epithelial ovarian cancer exhibited reduced erythrocyte Se level when compared to the healthy control group, as measured by an inductively coupled plasma mass spectrometer. According to these studies, Se level in blood is negatively correlated with ovarian cancer malignancy.

Overall, most of the research results in this section indicate the negative correlation between blood Se concentration and the occurrence of gynecological tumors, and blood Se concentration may also be associated with prognosis. However, the potential role of this biomarker in risk stratification and prognosis assessment still requires further investigation.

### 2.3 Tissue Se Concentration and the Occurrence of Gynecological Cancers

Different from the blood Se, the tissue Se level reflected long‐term Se intake. This section examines the association between Se level in human tissues and the risk of gynecological cancers, as investigated in case‐control studies.

Cunzhi et al. [27] measured Se content in cervical cancer tissues to explore the relationship between trace elements and the incidence of cervical cancer by atomic fluorescence spectrometry. These findings indicated that the Se content in cervical cancer tissues was significantly lower compared to the paired non‐lesion tissues. Wadhwa et al. [56] included 31 cervical cancer cases and evaluated the Se level in the scalp hair by atomic absorption spectrometry to reveal the role of Se in carcinogenic processes. Results showed that, compared with healthy volunteers, cervical cancer patients exhibited significantly lower Se level in scalp hair samples.

In tissue samples that were formalin‐fixed and paraffin‐embedded, including 20 malignant epithelial ovarian cancers, 15 epithelial borderline tumors, and 20 non‐neoplastic healthy ovaries, Canaz et al. [57] used atomic absorption spectroscopy to measure the Se content. However, the concentration of Se was not statistically different between them. Wadhwa et al. [56] enrolled 19 ovarian cancer cases and evaluated the Se level in the scalp hair by the same method. Compared with healthy volunteers, 51%–53% lower Se was observed in scalp hair in ovarian cancer patients.

### 2.4 Se Supplementation Therapy in Gynecological Cancer Prevention

Following oral administration of a sodium selenite (Na_2_SeO_3_) solution (276 µg Se daily), Kowalska et al. [58] observed that heterozygous carriers with deleterious BRCA1 gene mutations exhibited a decreased frequency of chromosome breaks compared to carriers who did not receive Se supplementation. Furthermore, Huzarski et al. [33] conducted a pilot investigation that enrolled 200 healthy BRCA1 mutation carriers, utilizing a matched‐pair design with 100 case‐control pairs. Following two years of oral Se administration using a Se solution manufactured by Vifarm S.A., as compared to women who did not take supplements, women taking supplements had a two‐fold lower risk of BRCA1‐associated ovarian cancer. The existing research revealed the potential preventive effect of Se supplementation therapy in BRCA1‐mutated ovarian cancer.

### 2.5 Se Supplementation Decreases the Toxicity of Chemotherapy and/or Radiotherapy for Gynecological Cancers

The adverse side effects caused by chemotherapy and radiotherapy may be reduced by Se supplementation in several studies [64, 65]. Yang et al. [59] conducted a randomized trial in which 104 patients diagnosed with FIGO stage IIB cervical cancer were allocated to receive either Se yeast tablets (200 µg Se daily) or matching placebo tablets. When receiving standard treatment, including pelvic external irradiation, concurrent chemotherapy, and brachytherapy, the Se supplementation group demonstrated a dramatic reduction in grade 3 myelosuppression and thrombocytopenia compared with patients who did not receive Se supplementation. Of note, the therapeutic effect of chemoradiotherapy was not compromised by Se supplementation. Muecke et al. [60] recruited 11 cervical cancer cases and 70 uterine cancer cases to either receive sodium selenite orally or not receive a supplement when treated by radiotherapy. External radiotherapy used three‐dimensional conformal radiotherapy. Throughout the radiotherapy course, participants in the Se supplementation group were administered 500 µg of Se on treatment days and 300 µg on non‐treatment days, continuing until the final radiotherapy session. The supplementation was provided as inorganic sodium selenite (Selenase, biosyn Arzneimittel GmbH). Notably, Se supplementation administered during radiotherapy significantly decreased both the incidence and severity of radiation‐induced diarrhea. Similarly, Assessment of overall survival (OS) and disease‐free survival (DFS) revealed no statistically significant differences between the control group and the Se‐supplemented group, suggesting that Se supplementation did not affect the therapeutic effect of radiotherapy.

Sieja et al. [61] enrolled 62 ovarian cancer patients receiving multidrug chemotherapy regimens that included cisplatin in combination with cyclophosphamide, and then divided them into the experimental group receiving the drug Protecton Zellactiv two capsules, four times daily (total Se 200 µg) and the control group without Se supplementation. The serum and hair concentration of Se increased significantly after Se supplementation. Additionally, following 2 and 3 months of Se supplementary treatment, ovarian cancer patients receiving chemotherapy showed significant increases in GSH‐P(x) activity in erythrocytes, platelets, and white blood cells. It's worth noting that a significant reduction in flatulence, weakness, malaise, loss of appetite, hair loss, and abdominal pain was stated in the experimental group.

Functioning as an optimal chemoprotective agent, Se supplement mitigated the adverse effects associated with chemotherapy and/or radiotherapy while preserving anticancer efficacy and demonstrating an absence of toxicity in patients. The Institute of Medicine (USA) has proposed for adult humans a recommended Se dietary allowance of 55–75 µg/day [66]. The supranutritional level of Se (200–500 µg per day) shows potential preventive effect in BRCA1‐mutated ovarian cancer and a protective mechanism that significantly reduces the toxicity and adverse side effects associated with chemotherapy and radiotherapy. However, there is no universally accepted definition for high‐dose Se in clinical translation and cancer therapy, despite several phase I clinical trials targeting advanced or refractory tumors. In these early‐stage trials, to achieve the pro‐oxidant effect, the short‐term pharmacological administration often reached the milligram level [67]. Inevitably, such elevated doses are accompanied by severe systemic toxicity (selenosis), which represents the most significant challenge for high‐dose Se therapy in clinical therapy [68].

## 3 The Therapeutic Application of Small‐Molecule Se Compounds in Gynecological Cancers

In recent years, more and more reports have proved the high therapeutic efficiency of Se‐based compounds in cancer treatment. Inorganic Se compounds possess cytotoxic properties, killing cancer cells directly and inhibiting their proliferation. Organic Se compounds play a prominent role in selenoprotein synthesis, which are absorbed, highly utilized, and easily stored in tissues. Thus, they tend to be used for prevention and auxiliary treatment [69]. In this section, the use of small‐molecule Se compounds in gynecological cancers treatment is summarized (Table 2).

**TABLE 2:** Summary of therapeutic applications of small‐molecule Se compounds in gynecological cancers.

Classification	Application	Compounds name	Cancer Types	Characteristics	Refs.
Inorganic Se compounds	Anti‐tumor effect	sodium selenite	cervical cancer	Active the AMPK/mTOR/FOXO3a pathway; Impair mitochondrial function	[70]
		sodium selenite	cervical cancer	Inhibit glucose metabolic reprogramming via ROS‐mediated inhibition of AKT/mTOR/HIF‐1α pathway	[71]
		sodium selenite	cervical cancer	Induce cells’ DNA damage; Active p53 and p38 pathway	[72]
		sodium selenite	cervical cancer	Augment ROS; Impair mitochondrial function; Active AMPK/FOXO3a/GADD45a axis	[73]
		sodium selenite	cervical cancer	Inhibit PI3K/AKT signaling pathway	[74]
		sodium selenite	cervical cancer	Augment ROS; Decrease mitochondrial membrane potential	[75]
		sodium selenite	ovarian cancer	Augment ROS; Reduce the level of selenoprotein GPx4	[76]
		SeO_2_	cervical cancer	Inhibit histone demethylase protein expression; Removing the trimethylation modification of histone H3K27	[77]
		SeO_2_	cervical cancer	Upregulate the levels of caspase‐3, p53 proteins, and cell death‐related microRNA let‐7a	[78]
		Ru‐Se	cervical cancer	Shuttle electrons from biological electron donors to cause oxidative stress	[79]
		Au‐Se, Ru‐Se, and Pd‐Se	ovarian cancer	Pro‐apoptotic effect	[80]
Inorganic Se compounds	Synergistic effects with radiotherapy or chemotherapy	sodium selenite	ovarian cancer	Reverse cisplatin resistance	[81]
		Ru‐Se	cervical cancer	Enhance the sensitization effect during X‐ray by inducing cellular sub‐G1 cycle arrest, mitochondrial damage, and DNA damage	[82]
		Ir‐Se	cervical cancer	Reverse cisplatin resistance via generating excessive singlet oxygen and down‐regulating mitochondrial membrane potential	[83]
Organic Se compounds	Anti‐tumor effect	MeSeA, MeSeCys, SeMet	cervical cancer	Inhibit ERK and AKT signaling pathways	[84]
		dGMPSe	cervical cancer	Release of H_2_Se	[85]
		sucrose‐Se	cervical cancer	DNA damage	[86]
		CMPS	ovarian cancer	Augment ROS	[87]
		2‐(4‐methylphenyl)‐1,3‐selenazol‐4‐one	ovarian cancer	Decrease the mitochondrial membrane potential and trigger the translocation of AIF into the nucleus	[88]
		SeCPS‐II	ovarian cancer	Active p53/Bax/caspase pathway	[89]
Organic Se compounds	Synergistic effects with radiotherapy or chemotherapy	SeCys	cervical cancer	Synergy with X‐ray increases ROS generation; active caspase‐3/‐8/‐9; Induce the lysis of PARP; Activate p53 and MAPK phosphorylation, as well as downregulation of p‐AKT and p‐ERK	[90]
		SeD	cervical cancer	Synergy with X‐rays; active p53 pathway by promoting the generation of ROS and inducing DNA damage	[91]

### 3.1 The Anti‐Tumor Effect of Inorganic Se Compounds in Gynecological Cancers

Consistent evidence from cervical cancer in vitro research demonstrated the dose‐ and time‐dependent suppression of HeLa and SiHa cell viability by sodium selenite, while also exhibiting significant antitumor efficacy in vivo [70, 71, 72, 73, 74, 75]. Mechanistically, Lv et al. [70] revealed that sodium selenite triggered autophagy and cell apoptosis by activating the AMPK/mTOR/FOXO3a pathway in HeLa and SiHa cells. Furthermore, sodium selenite induced mitochondrial dysregulation, characterized by diminished membrane potential, elevated Ca^2^^+^ overload, and augmented ROS generation. Zeng et al. [71] reported that sodium selenite suppressed glucose metabolic reprogramming in HeLa and SiHa cells via ROS‐mediated inhibition of AKT/mTOR/HIF‐1α pathway, consequently reducing cellular proliferation and migration while promoting apoptosis. Rudolf et al. [72] explored the mechanism of cervical cancer cell apoptosis induced by sodium selenite and found that sodium selenite induced HeLa cells’ DNA damage in a dose‐ and time‐dependent manner as well as p53 and p38 pathway activation. In Qi's study [73], sodium selenite increased intracellular ROS and impaired mitochondrial function, as well as enhanced the AMPK/FOXO3a/GADD45a axis. Consequently, this treatment reduced the viability of HeLa and SiHa cells, induced S‐phase cell cycle arrest, and promoted cellular apoptosis. Wang et al. [74] found that sodium selenite exerted its anti‐tumor effect by inhibiting cell viability, inducing cell apoptosis in HeLa and SiHa cells through suppressing the PI3K/AKT signaling pathway. Fu et al. [75] explored the differentially expressed proteins with the treatment of sodium selenite in HeLa cells. Approximately 1000 protein spots were detected, with 13 differentially expressed proteins subsequently identified through mass spectrometry analysis. The majority of these proteins were associated with oxidative stress pathways, including peroxiredoxins and superoxide dismutase. Moreover, sodium selenite treatment triggered a rise in ROS generation and a decline in mitochondrial membrane potential in HeLa cells.

Choi et al. [76] observed that high‐dose sodium selenite showed significantly inhibited SKOV3 cells’ growth and increased cells’ apoptosis, which was mediated via ROS augmentation. In vivo, intravenous high‐dose sodium selenite (2000 µg/kg thrice weekly for 2 weeks) significantly suppressed the subcutaneously transplanted tumor growth in SKOV3‐bearing mice. It was worth noting that high‐dose sodium selenite reduced the level of selenoprotein GPx4, a protein with antioxidant properties, which further triggered lipid peroxidation and ferroptosis.

According to Chen et al. [77], in both cervical cancer HeLa and SiHa cells, SeO_2_ suppression cells’ proliferative capacity as well as migration and invasion capabilities, while increasing cell apoptosis. In vivo, the administration of SeO_2_ solution intraperitoneally effectively suppressed the tumor growth in the subcutaneous xenograft mouse model established by SiHa cells. Mechanistically, SeO_2_ inhibited histone demethylase protein expression, especially JMJD3 and UTX, removing the trimethylation modification of histone H3K27. Liu et al. [78] found that SeO_2_ exerted significant dose‐dependent inhibitory effects on cell proliferation and viability, while concurrently inducing apoptosis in cervical cancer HeLa cells. This anti‐tumor pattern was mediated through the upregulation of caspase‐3 and p53 protein levels, along with increased expression of the cell death‐associated microRNA let‐7a.

Metal‐based compounds have been well‐documented in medicine. As a new therapeutic agent, it offers numerous advantages over traditional inorganic compounds. Due to its stronger nucleophilic power, Se can readily coordinate with soft metal ions, thereby forming metal complexes that have pharmacological effects [92]. In Qin's study [79], ruthenium (Ru) metal centers were used to coordinate with electrophilic centers to activate Ru‐Se complexes. The results demonstrated that Ru‐Se complexes effectively mediated electron transfer from biological electron donors, thereby inducing oxidative stress. The electron transfer rate at the Se electrophilic center in HeLa cells demonstrated a 1.81‐fold increase. Consequently, the Se electrophilic center exhibited a 14.98‐fold higher cytotoxicity against cervical cancer HeLa cells relative to normal cells.

Molter et al. [80] constructed aurum selenide (Au‐Se), Ru‐Se, and palladium selenide (Pd‐Se) and compared their anti‐tumor activities. Results showed that all these compounds with Se dose‐dependently reduced the proliferation of ovarian cancer cells A2780.

Overall, metallic Se compounds hold promise as the next‐generation therapeutics for the treatment of cancer.

### 3.2 The Synergy Effect of Inorganic Se Compounds With Radiotherapy or Chemotherapy in Gynecological Cancers

#### 3.2.1 Sodium Selenite

Caffrey et al. [81] investigated the capacity of Sodium selenite to reverse resistance development using a cisplatin‐resistant mouse model. In this experimental design, immunodeficient nude mice received subcutaneous injections of A2780 ovarian cancer cells. Upon reaching a tumor volume of 0.5 cm^3^, the mice received a low dose of cisplatin (2.6 mg/kg) via intraperitoneal injection. After 1 week, a second intraperitoneal injection of cisplatin with a 7.2 mg/kg dose was administered to test for sensitivity to cisplatin. The results showed that tumor volume in the cisplatin‐pretreated group was significantly larger compared to the PBS‐pretreated group, indicating the development of cisplatin resistance. After pre‐treatment with cisplatin (1.5 mg/kg each mouse), sodium selenite was injected intraperitoneally 24 h, 4 h before, and 24 h after, respectively. Interestingly, cisplatin resistance of mice was reversed by sodium selenite synchronized treatment. The primary tumor cells were then extracted and cultured for approximately 2 months. Colony formation was measured to determine whether the cells were sensitive to cisplatin. Results showed that the cell proliferation ability in the cisplatin + sodium selenite pretreatment group with cisplatin treatment in vitro was lower than that of the cisplatin‐pretreatment group. Mechanically, cisplatin + sodium selenite pretreatment reversed the cisplatin resistance by preventing the increase of glutathione (GSH) levels induced by cisplatin. Consequently, sodium selenite has the capacity to counteract platinum‐induced resistance in ovarian cancer cells [93].

#### 3.2.2 Metallic Selenium Compounds

Cao et al. [82] developed a Se‐based Ru(II) metal complex (Ru‐Se) that exhibited cytochrome P450 enzyme‐mimetic properties. The combination of Ru‐Se with radiotherapy triggered sub‐G1 phase cell cycle arrest, mitochondrial impairment, and DNA damage‐mediated apoptosis in HeLa cells. Consequently, Se complex significantly potentiated the radiosensitizing effect during X‐ray radiotherapy. Huang et al. [83] compounded a novel Se‐based iridium(III) complex (Ir‐Se), which was synthesized by conjugating an aromatic Se ligand with an iridium(III) complex via a straightforward imine formation reaction. Through endocytosis, Ir‐Se entered cisplatin‐resistant HeLa cells and then induced cell apoptosis by generating excessive singlet oxygen and down‐regulating mitochondrial membrane potential, which exhibited high cytotoxicity to reverse cell resistance to cisplatin. Of note, the cisplatin resistance index (RI) of HeLa cells was largely reduced from 27.5 to 5.6 after Ir‐Se complex treatment, indicating the ability of Ir‐Se to overcome cisplatin resistance in cervical cancer.

### 3.3 The Anti‐Tumor Effects of Organic Se Compounds in Gynecological Cancers

Study reported that natural organic Se compounds methylseleninic acids (MeSeA), MeSeCys, and SeMet significantly inhibited the proliferation, migration, and adhesion mediated by the suppression of ERK and AKT signaling in HeLa cells [84]. Krakowiak et al. [85] synthesized a new Se derivative, 2'‐deoxyguanosine‐5'‐O‐selenophosphate (dGMPSe). The cytotoxicity of dGMPSe was caused by the release of H_2_Se, which could effectively kill cervical cancer HeLa cells. Guo et al. [86] synthesized sucrose‐Se by the reaction of sucrose with Se trichloride, which led to the dose‐dependent suppression of HeLa cells’ proliferation and concomitantly triggered cell apoptosis.

The carboxymethyl cottonin Se (CMPS) was synthesized using carboxymethyl cottonin (CMP) as the substrate by the response surface method. The CMPS treatment inhibited proliferation of ovarian cancer A2780 cells, enhanced ROS production, and induced cell apoptosis [87]. Ahn et al. [88] synthesized a Se compound, 2‐(4‐methylphenyl)‐1,3‐selenazol‐4‐one, and found that it induced cell apoptosis in ovarian cancer SKOV3 cells. In Sun's study [89], Se‐rich C. *gunnii*. was constructed via liquid fermentation by the combination of C. *gunnii* with sodium selenite. Following the extraction of Se‐enriched *C. gunnii*, a new Se‐polysaccharide (SeCPS‐II) was isolated. SeCPS‐II exerted a dose‐ and time‐dependent inhibition on the viability of SKOV3 cells. In vivo within an ovarian in situ tumor model in rats established by SKOV3 cells, SeCPS‐II significantly inhibited tumor growth. Mechanistically, SeCPS‐II significantly inhibited SKOV3 cells’ viability and stimulated cell apoptosis of through the p53/Bax/caspase pathway.

### 3.4 The Synergy Effect of Organic Se Compounds With Radiotherapy or Chemotherapy in Gynecological Cancers

SeCys is an analogue of cystine (Cys). Xie et al. [90] found that compared with the treatment of X‐ray or SeCys alone, the combination therapy led to a significant suppression in cell viability of HeLa cells. Moreover, the ROS level and radiation‐induced cell apoptosis showed a higher increase with the combination therapy, which might be attributed to the Auger electron effect induced in Se atoms upon exposure to X‐ray radiation. Mechanistically, the combination of X‐ray irradiation and selenocysteine (SeCys) treatment triggered the activation of caspase‐3/‐8/‐9 in HeLa cells, which subsequently led to the cleavage of poly‐ADP‐ribose polymerase (PARP). In addition to DNA damage, excessive ROS production activated p53 and MAPK phosphorylation, as well as downregulation of p‐AKT and p‐ERK, ultimately increasing radiation sensitivity and inhibiting tumor proliferation. Our group [91] previously conducted a collaborative screening of different types of Se compounds (with valence states ranging from ‐2, 0, +4, and +6) to identify the potential radiosensitization effect on cervical cancer. Results showed that organic Se compounds, selenodiazole derivative (SeD), exhibited a stronger anti‐cancer effect and had a higher safety index. Combined with X‐rays synergistically, it inhibited cell growth, clone formation, migration, and arrested the G2/M phase, while triggering cell apoptosis. Additional mechanistic investigations have demonstrated that SeD facilitated p53 pathway activation through the promotion of ROS generation, subsequently triggering DNA damage. SeD combined with X‐ray irradiation markedly suppressed hepatic metastasis in cervical tumor‐bearing mice, while simultaneously mitigating the adverse effects associated with radiotherapy. Organic Se could therefore be further developed as a less toxic and more effective radiosensitizer for cervical cancer.

### 3.5 Comparative Antitumor Mechanisms of Small‐Molecule Selenium Compounds in Gynecological Cancers

In Gynecological cancers, both inorganic and organic Se compounds can induce oxidative stress, trigger DNA damage, impair mitochondrial function, and ultimately activate apoptosis. Both also demonstrate the ability to reverse chemotherapy resistance and sensitize cells to radiotherapy. However, different Se compounds trigger diverse modes of cell death. Organic Se compounds are typically considered less toxic than their inorganic counterparts, as they must undergo metabolic transformation before producing cytotoxic effects [94]. For instance, inorganic Se compounds, including sodium selenite, are typically converted to HSe^−^. Under conditions where thiols and oxygen are available, selenide can facilitate the production of superoxide and other ROS. The accumulation of ROS disrupts mitochondrial permeability transition pores, which promotes cytochrome c release and ultimately induces apoptosis [95]. By comparison, most organic Se compounds are commonly metabolized to methylselenol, which generally does not promote superoxide generation. Instead, it primarily triggers caspase‐dependent apoptotic pathways and may lead to DNA fragmentation [91, 93, 94, 96]. Therefore, elucidating the cytotoxic mechanisms of various Se compounds may facilitate the rational design and clinical application of Se‐based therapeutics in gynecological cancers.

## 4 Characteristics of Se‐Based Nanoparticle

The semiconductor Se exhibits excellent photovoltaic, electrical conductivity, and photoconductive properties, as well as exceptional oxidative properties and thermoelectric responses [97]. Nanoparticle based on Se have attracted increasing attention in biomedicine due to their unique properties [98, 99]. To provide a theoretical basis for the application of Se‐based nanoparticles in gynecological cancers therapy and diagnosis, this section describes the physicochemical and biological properties of Se‐based nanoparticles.

### 4.1 Physicochemical Properties

Nanoparticles with Se^2−^: This type of Se nanoparticles is mainly derived from metallic Se compounds. Selenide nanoparticles such as iron selenide (Fe‐Se), copper selenide (Cu‐Se), cadmium selenide (Cd‐Se) nanoparticles, etc. The properties of metal selenide nanomaterials are enhanced by the properties of Se combined with the ions of individual metals, including high stability, catalytic activity, outstanding photothermal performance, sensitive responsiveness, functionalization ease, and lower toxicity [100]. The composition and morphology of metallic selenide nanomaterials could be controlled using optimized synthesis routes, new preparation methods, and improved reaction conditions. Metal selenide nanomaterials, additionally, have been extensively employed in tumor therapy owing to their exceptional near‐infrared light absorption capacity, superior photothermal conversion efficiency, and outstanding thermal stability. They may also produce toxic ·OH through Fenton or Fenton‐like reactions when mixed with effective concentrations of H_2_O_2_ [100].

0‐valent selenium nanoparticles (SeNPs) represent the most classical form of elemental Se nanoparticles, synthesized from inorganic Se through chemical processes or biosynthesis. These nanoparticles serve multi‐functions such as drug delivery vehicles and therapeutic agents, particularly when combined with radiotherapy or chemotherapy regimens [96]. SeNPs’ physical and chemical properties depend on their preparation method as well as the compounds used for their stabilization and functionalization. Freshly prepared naked SeNPs would aggregate into large clusters and cannot be stored for over 1 day [101]. There have been several compounds that have been used to stabilize SeNPs, such as amino acids, bovine serum albumin (BSA), polysaccharides such as chitosan and gum arabic, and polymers such as poly lactic‐co‐glycolic acid (PLGA) [102]. Owing to their intermediate valence state, the SeNPs possessed the capacity to act as both an electron donor and an acceptor in intracellular redox reactions. To elucidate the mechanism driven by the intracellular environment, Xiong et al. [103] fabricated 0‐valent Se nanoclusters (SeClus) through the assembly of engineered Se‐Se/Se‐S bonds. The strongly oxidizing environment of cancer cells oxidized intracellular 0‐valent Se to SeO_3_^2^^−^, yielding a cytotoxic species that inhibited cellular proliferation. It has been reported that SeO_3_^2^^−^ induced free radical accumulation, thereby exerting greater killing effects toward cancer cells [104].

Nanoparticles with Se^4+^: By enhancing secondary electron transport under X‐ray radiation through the electron transfer chain, high‐valent Se nanoparticles have strong electron transfer and redox disturbance to simultaneously enhance physical and biochemical potentiation in cancer treatment [105].

### 4.2 Biological Properties

#### 4.2.1 Metabolism and Biodegradability

Se‐based nanoparticles with different Se valence states can be gradually redox and transformed into the biologically active form of Se in the body. For example, in our previous study, MoSe_2_ nanosystems with Se^2−^ were metabolized into SeCys_2_ and MeSeCys in cervical cancer HeLa cells, and further synthesized selenoproteins SelO, SelT, and GPx4 [106].

Nanoparticles based on Se (0) are mostly oxidized to be cytotoxic SeO_3_^2−^ in the form of Se^4+^ in the oxidizing microenvironment of cancer cells [103]. Se^4+^ is further reacted into selenodiglutathione (GSSeSG), selenenylsulfide (GSSeH) [107]. While Se (0) mainly reacts with NADPH/GR/GSH and cysteine (CySH) to form selenocysteine (SeCys_2_) with Se^2−^ in normal cells [103]. SeCys_2_ is reduced to SeCys by glutathione and glutathione reductase, and SeCys is converted to H_2_Se under the action of selenocysteine β‐lyase and used for the synthesis of selenoprotein [107]. Therefore, H_2_Se is an intermediate product of the metabolism of Se, which is involved in selenoprotein synthesis after activation to selenophosphoric acid [107]. A major small‐molecule metabolite of Se is dimethyl selenide (DMSe) found in breath, trimethylselenonium (TMSe^+^) found in urine, and selenosugar, a metabolite that is found in tissues, urine, and feces. Moreover, Se can also be transported from hepatocyte to other cells in the form of selenosugar [108, 109].

Hu et al. [110] revealed that after high‐valence Se nanoparticles with Se^4+^ treatment, Se metabolite levels of SeCys_2_, MetSeCys, and Se^4+^ were significantly higher in tumor tissues.

**FIGURE 3:**
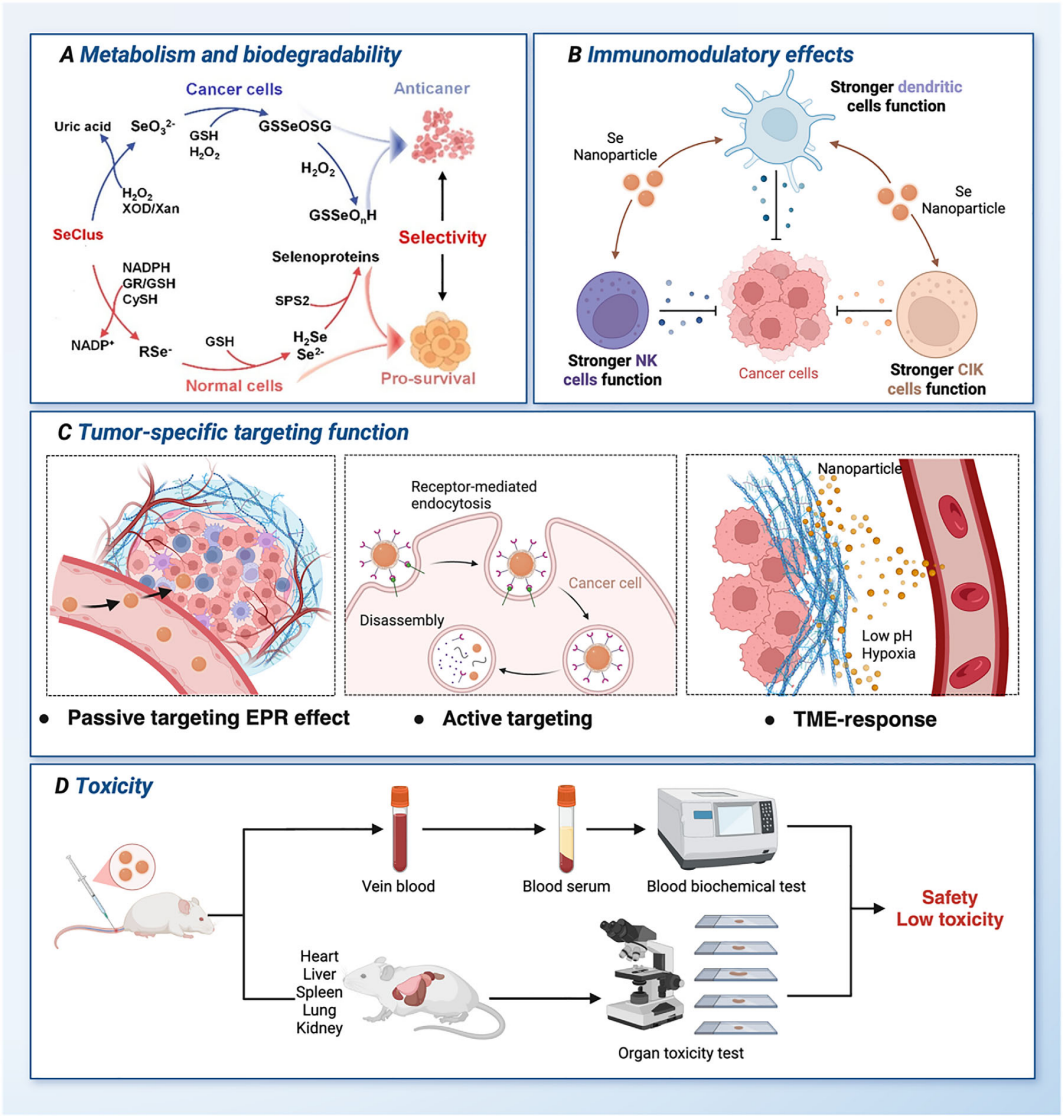
Biological properties of Se‐based nanoparticles in gynecological cancers. (A) Metabolism and biodegradability properties. (B) Immunomodulatory effects. (C) Tumor‐specific targeting function. D) The toxicity of nanoparticles. Part A was reproduced with permission. [103], Copyright 2024, John Wiley and Sons/Wiley‐VCH. (Others were created with BioRender.com).

On the whole, Se‐based nanoparticles have degradability. Future research needs to pay more attention to the metabolic process of Se‐based nanoparticles in the body (Figure 3A).

#### 4.2.2 Immunomodulatory Effects

Nanobiomaterial‐based strategies that are being used to target and modulate immune cells [111]. It has been demonstrated that functional Se nanoparticles can be used for immunotherapy, achieving a higher level of treatment efficiency as adjuncts to current cancer treatments [112]. For example, Se‐based nanoparticles could significantly potentiate the cytotoxicity of cytokine‐induced killer cells (CIK) against tumor cells through the modulation of selenoprotein expression or promoting actin acetylation to facilitate the elimination of tumor cells through adaptive immune cells such as γδT cells [113]. In addition, SeNPs significantly increase the maturation of dendritic cells (DCs) in lymph nodes. Lentinan‐SeNPs effectively activated natural killer cells (NK) and enhanced the phagocytic ability of macrophages to exert anti‐tumor effects [114], indicating the essential role of Se‐based nanoparticles in activating the innate and adaptive immune systems (Figure 3B).

#### 4.2.3 Tumor‐Specific Targeting Function

The targeted delivery of nanomedicines is divided into passive targeting, active targeting, and stimuli‐responsive targeting systems, such as pH‐responsive, hypoxia‐responsive, etc. [48] (Figure 3C).

A tumor's physiological properties facilitate passive targeting, which improves the nanoparticle‐based delivery systems accumulation [115]. The rapid growth of tumors results in increased vascular permeability and compromised lymphatic drainage, leading to the collective enhancement of the nanosystem accumulation via the enhanced permeability and retention (EPR) effect [116]. Nevertheless, the heterogeneous effect of the EPR throughout various tumor tissue regions frequently results in variable delivery efficiency, which is contingent upon the tumor types and developmental stages.

Active targeting involves the targeting ligands of cancer binding to receptors on cell membranes, which will increase the selective uptake of nanoparticles in tumor tissue and reduce the toxic effect on normal cells [117]. For example, Se nanoparticles modified by hyaluronic acid (HA) achieved nanoscale targeting by binding to CD44 via the HA surface [117]. Folate‐conjugated Se nanoparticles (FA‐SeNPs) showed significantly improved cellular uptake through FA‐mediated endocytosis via both nystatin‐dependent lipid raft‐mediated and clathrin‐mediated pathways [118].

In recent years, anti‐cancer nanotherapeutics that respond to tumor microenvironments (TMEs) have gained considerable attention due to the rapid development of nanotechnology in biomedicine [119]. Using the features of TMEs, functionalized Se nanosystems can be activated under specific conditions, enabling spatiotemporal modulation of TMEs [48]. For example, TMEs with low pH could release drugs rapidly under certain oncological conditions [48]. Luesakul et al. [120] designed a doxorubicin (DOX)‐loaded SeNPs@TMC‐FA, which exhibited an accelerated release profile under acidic conditions in ovarian cancer OVCAR8 cells. Additionally, hypoxia is a common feature of TMEs. Hypoxia‐responsive nanoparticles show enhanced drug delivery efficacy through hypoxia‐induced drug release mechanisms, prolonged circulation time, and high drug accumulation in the tumor [121].

#### 4.2.4 Toxicity

The chemical form of Se‐based nanoparticles present is a crucial factor in determining their toxicity. SeNPs are biocompatible and less toxic than selenite and selenate [122]. Among SeNPs, stabilized SeNPs are less toxic than bare SeNPs [102]. In addition, Se concentration is also an important factor affecting its toxicity. At low concentrations, it acts as an antioxidant, which balances redox homeostasis and reduces the effect of oxidative stress caused by ROS on normal cells [21]. In fact, the toxicity of Se‐based nanoparticles is selective in cells. In the reductive environment of normal cells, Se serves as a fundamental substrate for selenocysteine formation and selenoprotein synthesis, thereby supporting essential cellular functions [103]. Therefore, Se‐based nanoparticles have relatively low toxicity to normal cells.

Additionally, nanomaterials based on Se were evaluated for their biocompatibility in vivo experiments. As a result of comprehensive in vivo experiments, including our group [110, 123, 124], Se‐based nanomaterials did not change mice weight during the treatment period, indicating a favorable biosafety. Compared to the control group, histological images revealed no observable organ damage in mice treated with Se‐based nanomaterials, indicating no obvious toxicity. Among mice in each treatment group, all blood biochemical indices were within normal limits, including hepatic function parameters (aspartate aminotransferase [AST], alanine aminotransferase [ALT]), renal function markers (urea [UREA], uric acid [UA], creatinine [CREA]), glycemic indicators (glucose [GLU]), and lipid profile components (cholesterol [CHO]), etc., further suggesting the high safety of Se‐based nanomaterials treatment strategy (Figure 3D).

## 5 Therapeutic Applications of Se‐Based Nanoparticles in Gynecological Cancers

A variety of nanomaterials are being developed for gynecological cancers treatment based on the unique physical and chemical properties of Se‐based nanoparticles. In the following section, the anti‐tumor effects of Se‐based nanoparticles, including direct killing, radiotherapy sensitization, chemotherapy drug delivery, chemotherapeutic sensitization, combined with photothermal therapy, and chemodynamic therapy, are reviewed (Table 3).

**TABLE 3:** Summary of therapeutic applications of Se‐based nanoparticles in gynecological cancers.

Applications	Nanoparticles	Cancer Types	Characteristics	Refs.
Directly kill cancer cells	MSP‐SeNPs	cervical cancer	Eliminate DPPH and ABTS free radicals	[125]
	CPA‐SeNPs	cervical cancer	Augment ROS; Activate caspase‐3 mediated apoptosis; Induce mitochondrial dysfunction	[126]
	SA‐Se‐NPs	cervical cancer	Activate caspase‐3 and PARP cleavage mediated apoptosis	[127]
	CT@SeNPs	cervical cancer	Cytotoxicity	[128]
	Jm/SeNPs	cervical cancer	Cytotoxicity	[129]
	RGDfC‐SeNPs	cervical cancer	Augment ROS; Induce mitochondrial dysfunction	[130]
	RuSeNPs	cervical cancer	Induce DNA damage; Activate p53 mediated apoptosis; reduce mitochondrial membrane potential	[79]
	TQ‐SeNPs	endometrial cancer	Inhibit ERK2, MEK2, NFKB (p65) expression	[131]
	R‐SeNPs	ovarian cancer	Augment ROS	[132]
	LNT‐SeNPs	ovarian cancer	Drug delivery pathway mediated by the TLR4/TRAF3/MFN1 axis; Reduce IL‐1β, IL‐6 and TNF‐α secretion	[133]
	SeNPs	ovarian cancer	Increase the methylation of histone3 at lysine K9 and K27	[134]
	MUC16‐SeMnf@Res	ovarian cancer	Augment ROS; Deplete GSH; Induce mitochondrial dysfunction and inhibit MUC16 expression	[110]
	MBs@Cu‐Se NPs	ovarian cancer	Ultrasound‐responsive drug release	[135]
Combine with radiotherapy	PEG‐SeNPs	cervical cancer	Induce DNA damage; Activate caspase‐3 mediated apoptosis	[136]
	SeD@MSNs‐FA	cervical cancer	Augment ROS; Activate p53, AKT and MAPK mediated apoptosis	[137]
	MoSe_2_ NF‐RGD	cervical cancer	Augment ROS; Damage mitochondria; Upregulate the expression of selenoprotein TrxR2, GPx4, SelO and SelT. Activate immunogenic cell death	[106]
	CCMT	cervical cancer	Show synergistic anti‐tumor effect with X‐ray	[123]
Combine with chemotherapy	SeNPs@LNT	cervical cancer	Induce DNA damage; Decrease ABC transporters and VEGF expression	[124]
	FA‐SeNPs	cervical cancer	Activate clathrin‐mediated endocytosis; Activate p53, caspase‐3 mediated apoptosis	[138]
	PTX‐chit‐SeNPs	cervical cancer	Enhance Bax gene expression	[139]
	PTX‐SeNPs	cervical cancer	Augment ROS disrupting mitochondrial membrane potential; Activate caspases mediated apoptosis	[140]
	[W5R4C]‐SeNPs	ovarian cancer	Improve the cellular drugs uptake	[141]
Combine with photothermal therapy	Au@Se NPs	cervical cancer	Photothermal properties; augment ROS; Facilitate cleavage of caspase‐3/‐7/‐9 and PARP; Activate caspase‐mediated apoptosis; Activate immunogenic cell death	[142]
	Bi_2_Se_3_@PDA /DOX/HAS NPs	cervical cancer	Photothermal properties	[143]
	MoSe_2_ NDs	cervical cancer	Photothermal properties	[144]
Combine with chemodynamic therapy	SSMA/DOX	cervical cancer	Augment ROS; Catalyze endogenous H_2_O_2_ to ·OH for CDT	[145]

### 5.1 Directly Kill Cancer Cells

Based on the sodium selenite and ascorbic acid redox system, Shi et al. [125] used the six‐part polysaccharide (MSP) of *Morchella* as a novel stabilizer for the synthesis of SeNPs to MSP‐SeNPs. MSP‐SeNPs exhibited potent scavenging efficacy against both DPPH and ABTS free radicals. In vitro and in vivo experiments showed that it potently inhibited the growth of cervical cancer HeLa cells. Li et al. [126] synthesized water‐soluble dispersed Se nanoparticles (CPA‐SeNPs) by the redox reaction of 1,6‐α‐D‐glucan (CPA) and Na_2_SeO_3_ with ascorbic acid. Cell proliferation was significantly reduced by CPA‐SeNPs in vitro, which meanwhile induced cell apoptosis and arrested the S‐phase by producing excessive ROS, disrupting mitochondria, and activating caspase‐3. In addition, in Zheng's study [127], Sialic acid (SA) surface‐modified Se nanoparticles were synthesized (SA‐Se‐NPs). Surface modification with SA markedly improved both cellular uptake and cytotoxicity of Se nanoparticles. Moreover, SA‐Se‐NPs activated caspase‐3 and caused PARP cleavage to induce cell apoptosis in HeLa cells.

Hemanth et al. [128] synthesized the bifunctional citral‐tryptamine fused Se nanosphere (CT@SeNPs). Results showed that CT@SeNPs decreased the viability of cervical cancer cells SiHa in both time‐ and dose‐dependent manners. Besides, El‐Sherbiny et al. [129] reported the biosynthesis of Se nanoparticles (SeNPs) using Corchorus olitorius mucilage (Jm). Then, Jm/SeNPs and chitosan nanoparticles (Cht) were combined to form the bioactive nanocomposites (NCs). NCs induced significant apoptosis in HeLa cells, which was shown as the forms of apoptotic indices, cell shrinkage, cytoplasmic vacuolization, membrane blebbing, and cell debris. By loading biocompatible Se nanoparticles (SeNPs) with RGDfC peptides, Xia et al. [130] synthesized the tumor‐targeted gene delivery vector RGDfC‐SeNPs. RGDfC‐SeNPs were then uploaded with Derlin1‐siRNA to synthesize RGDfC‐Se@siRNA. A greater proportion of RGDfC‐Se@siRNA was absorbed by HeLa cells than by human umbilical vein endothelial cells. In vitro analyses revealed that RGDfC‐Se@siRNA suppressed cell invasion, migration, and proliferation while simultaneously promoting cell apoptosis in HeLa cells. RGDfC‐SeNPs@siRNA also showed pronounced anti‐tumor activity in a mouse model with subcutaneously transplanted tumors. In terms of mechanisms, treatment with RGDfC‐Se@siRNA induced mitochondrial dysfunction in HeLa cells, characterized by elevated ROS levels and reduction of the mitochondrial membrane potential. In Qin's study [79], by utilizing targeted polymer surfactants at the nanoscale, the RuSeNP complexes exhibited preferential accumulation in cancer cells, which consequently lowered their toxic effects on non‐malignant cells. Compared with RuSe, nanoscale RuSeNPs had greater cytotoxicity to HeLa cell lines. RuSeNPs were more easily taken up by HeLa cells, while accumulating less in Ect1/E6E7, the normal cervix cells. Therefore, RuSeNPs exhibited cancer cell targeting properties. Mechanistically, after intercepting and translocating bioelectrons from donor molecules in cancer cells, RuSeNPs generated large amounts of superoxide anions, which damaged DNA and lowered mitochondrial membrane potential, inducing apoptosis through the p53 signaling pathway.

Gulbay et al. [131] designed a thymoquinone (TQ)‐encapsulated Se nanoparticles (TQ‐SeNPs). Endometrial cancer HEC1B cell activities were inhibited by TQ‐SeNPs with responses proportional to both temporal exposure and dosage intensity by downregulating ERK2, MEK2, and NFKB (p65) mRNA expression.

**FIGURE 4:**
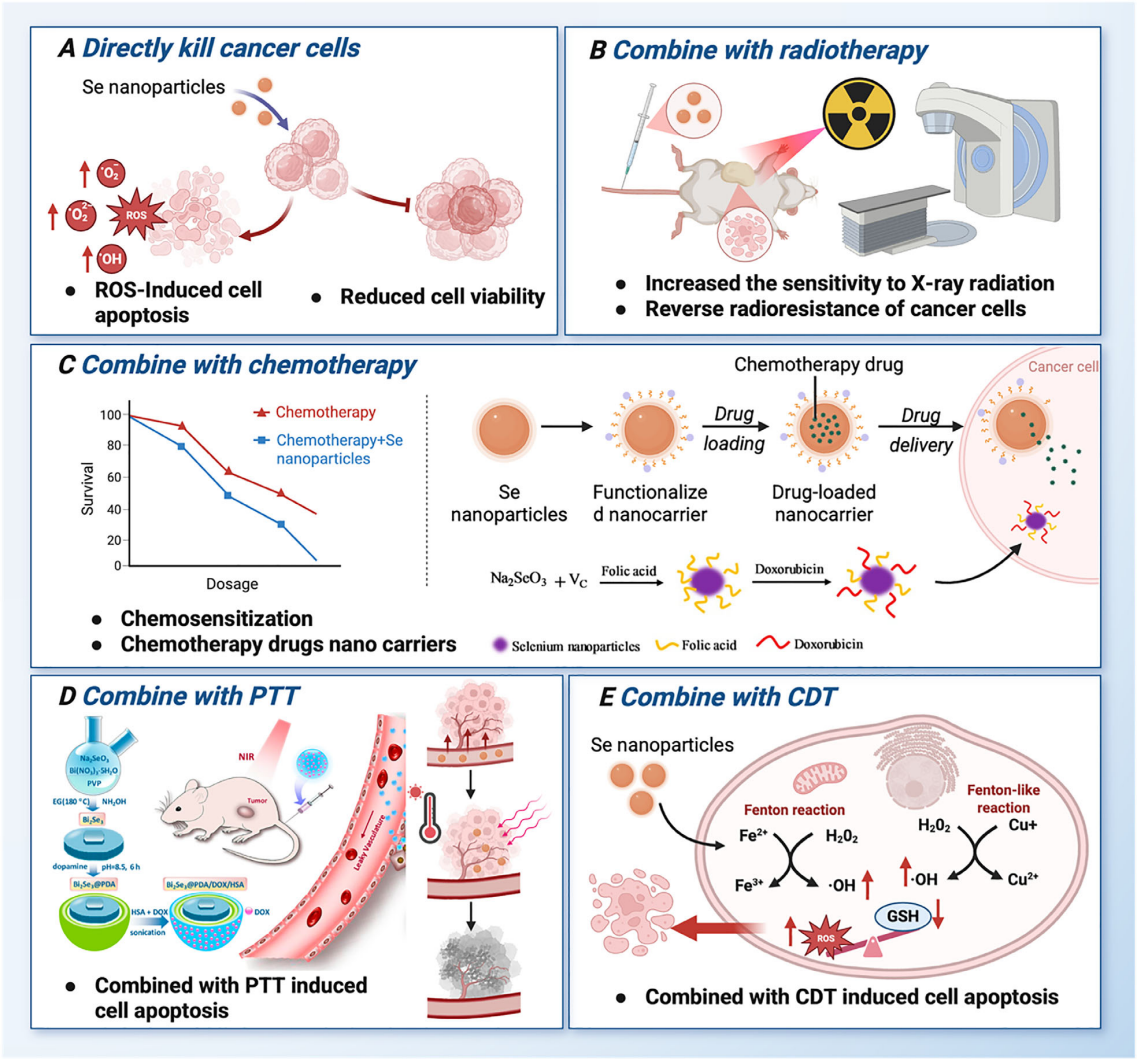
The therapeutic applications of Se‐based nanoparticles in gynecological cancers. (A) Directly kill cancer cells. (B) Combine with radiotherapy. (C) Combine with chemotherapy. (D) Combine with photothermal therapy. (E) Combine with chemodynamic therapy. Part C from ref. [138], Copyright 2018 by the authors, open access article. Part D was reproduced with permission. [143], Copyright 2015, American Chemical Society. (Others were created with BioRender.com).

As described by Wang et al. [132], RGDfC peptides were conjugated to the surfaces of SeNPs to fabricate siRNA delivery carriers (R‐SeNPs). Subsequently, MEF2D‐siRNA was conjugated to the surface of the carriers, resulting in the formation of functionalized Se nanoparticles designated as R‐Se@MEF2D‐siRNA. Firstly, this nanosystem was internalized by ovarian cancer SKOV3 cells via clathrin‐mediated endocytosis. Compared with the normal physiological environment, these functionalized Se nanoparticles released MEF2D‐siRNA more rapidly in the lysosomal microenvironment of tumor cells and inhibited SKOV3 cells’ proliferation and further induced cell apoptosis by excessive production of ROS. In vivo experiments also verified its anti‐tumor effect. Liu et al. [133] prepared lentinan (LNT) functionalized Se nanoparticles. In vitro assays showed that LNT‐SeNPs were mainly targeted mitochondria of tumor cells via cave‐mediated endocytosis and a drug delivery pathway mediated by the TLR4/TRAF3/MFN1 axis and induced apoptosis of ovarian cancer OVCAR‐3 cells. In ovarian ascites BALB/c nude xenografts via intraperitoneal injection of OVCAR‐3 cells, LNT‐SeNPs reduced the ascites volume and the number of cancer cells in the ascites compared to control. In addition, LNT‐SeNPs effectively induced apoptosis of cancer cells in abdominal ascites, and the secretion of pro‐inflammatory cytokines, such as IL‐1β, IL‐6, and TNF‐α, was also significantly inhibited. In another study, in the 3D spheroid tumor model of SKOV3 and OVCAR cells, both BSA and chitosan‐coated SeNPs treatment led to a significant reduction in cell viability compared to sodium selenite treatment. Specifically, Coated SeNPs induced enhanced histone methylation at lysine residues K9 and K27, activated lysine methyltransferase, and reduced S‐adenosylhomocysteine levels, which subsequently activated gene expression involved in biological pathways and processes such as Bcl‐2, P21, and wnt inhibitory factor 1 (WIF1) [134]. Hu et al. [110] designed a targeted therapeutic high‐valent Se nanomedicine (MUC16‐SeMnf@Res), which could target MUC16 to recognize ovarian cancer cells and simultaneously inhibit MUC16 protein expression, thereby achieving efficient treatment of ovarian cancer. In vivo experiments also verified its targeting properties and anti‐tumor effect. Mechanistically, the ratio of high‐valent Se^4+^ and increased Mn^2+^ in SeMnf disrupted intracellular redox homeostasis by inducing GSH depletion and excessive ROS production in SKOV3 cells. In addition, loading with resveratrol (Res) further amplified the impact of excessive ROS production, significantly inducing mitochondrial dysfunction and suppressing MUC16 expression, thereby promoting apoptosis and migration inhibition. Han et al. [135] designed the Cu‐Se nanoparticles coated with microbubbles (MBs) (MBs@Cu‐Se NPs). In ovarian cancer A2780 and CisRA2780 cells, MBs@Cu‐Se NPs exhibited a dose‐dependent reduction in cell viability (Figure 4A).

### 5.2 Combine With Radiotherapy

In cervical cancer radiotherapy, Yu et al. [136] synthesized the X‐ray responsive SeNPs using polyethylene glycol as the surface decorator (PEG‐SeNPs). Specifically, PEG‐SeNPs combined with radiotherapy significantly inhibited cell growth by inducing DNA fragmentation and caspase‐3‐mediated cell apoptosis. SeD@MSNs‐FA nanosystem designed by Liu et al. [137], which consisted of multifunctional mesoporous silica nanoparticles (MSNs) as carriers of SeD, with surface modification by FA to enhance targeting toward cervical cancer cells. Throughout the course of radiotherapy, SeD@MSNs‐FA enhanced cellular apoptosis and induced S‐phase arrest via the death receptor‐mediated apoptotic pattern, while suppressing the proliferation of HeLa cells. Thus, cervical cancer cells became more sensitive to X‐ray radiation. In vivo, in combination with X‐ray irradiation, SeD@MSNs‐FA effectively inhibited the growth of xenograft tumors in nude mice. Notably, the synergistic effect of SeD@MSN‐FA combined with X‐ray irradiation markedly enhanced intracellular ROS overproduction and induced apoptosis through regulation of the AKT, p53, and MAPK signaling pathways. Using MoSe_2_ nanomaterials, our group previously designed two radiosensitized nanosystems, namely two‐dimensional nanosheets (MoSe_2_ NS‐RGD) and three‐dimensional nanoflowers (MoSe_2_ NF‐RGD). The narrow band gap and excellent photoelectric/photothermal conversion properties of MoSe_2_ make it an attractive therapeutic nanoplatform for cancer radiotherapy. The combination of MoSe_2_ NF‐RGD and X‐ray had a superior synergistic therapeutic effect in HeLa and SiHa cells compared to MoSe_2_ NS‐RGD and control groups. Moreover, this synergy therapy led to excessive ROS production and exacerbated mitochondrial damage, resulting in irreversible DNA damage, which could be explained by a greater photocurrent response to X‐ray exposure. In vivo, MoSe_2_ NF‐RGD combined with X‐ray irradiation treatment markedly inhibited tumor growth compared to MoSe_2_ NS‐RGD and other treatment groups. Mechanistically, when MoSe_2_ NF‐RGD was transferred from lysosomes to the cytoplasm, it was first metabolized into SeCys_2_ and MeSeCys, and simultaneously caused mitochondrial damage and upregulated the expression of selenoprotein TrxR2 in mitochondria. Utilizing cervical cancer mouse models, MoSe_2_ nanosystems were further investigated for their robust radiosensitization properties. Interestingly, the combination of MoSe_2_ NF‐RGD and X‐ray irradiation significantly promoted dendritic cell maturation. Additionally, M2 macrophage (CD206^+^ F4/80^+^) proportion was significantly downregulated in tumor tissues with the treatment of MoSe_2_ NF‐RGD + X‐ray, whereas no such effect was observed with MoSe_2_ NS‐RGD plus X‐ray treatment. By establishing the bilateral subcutaneous xenotransplant tumor model, MoSe_2_ NS‐RGD + X‐ray application released tumor antigen and stimulated a stronger immune response in situ tumors, which significantly inhibited the growth of distant tumors via immunogenic cell death (ICD) [106]. In another study from our group, Cu_2‐x_Se nanomedicine (CCMT) camouflaged with TRAIL‐overexpressing cervical cancer cell membranes was designed. Given that the CCMT was encapsulated with TRAIL‐functionalized cell membranes, it demonstrated high targeting specificity toward cervical cancer cells, particularly X‐ray‐resistant cells exhibiting DR5 membrane receptor overexpression. Specifically, CCMT and X‐ray showed a synergistic inhibitory effect in HeLa X‐ray resistance (XR) cells in a dose‐dependent manner, demonstrating that the combination of CCMT with X‐ray irradiation exhibited enhanced efficacy in reversing radioresistance in cervical cancer cells. In addition, in HeLa‐XR cells, CCMT and X‐ray effectively suppressed cell proliferation, promoted apoptosis, and induced cell cycle arrest. In vivo, combined CCMT and radiotherapy markedly improved the antitumor efficacy of X‐ray, resulting in a significantly inhibitory of tumor volume than that observed in CCMT or radiotherapy monotherapy [123] (Figure 4B).

### 5.3 Combine With Chemotherapy

Our previous study [124] revealed that the viability of cervical cancer HeLa cells reverted and relapsed during cisplatin withdrawal. SeNPs@LNT in the cisplatin drug‐free interval prevented tumor recurrence via enhancing DNA damage and mitochondrial potential shifts. Then the in vivo investigation revealed significant suppression of tumor development following SeNPs@LNT treatment after cisplatin withdrawal. From the standpoint of mechanisms, SeNPs@LNT reduced drug resistance via decreasing ATP‐Binding Cassette (ABC) transporters and vascular endothelial growth factor (VEGF) expression in cervical cancer, thereby demonstrating the efficacy of Se nanoparticles in inhibiting cervical cancer progression during cisplatin‐free intervals. Based on the tumor‐targeting property of nanoparticles, they were often used as carriers for delivering chemotherapy drugs. For example, Xia et al. [138] synthesized the delivery vector FA‐SeNPs nanoparticles targeting tumors and loaded the anti‐cancer drug DOX onto their surface. Then, FA‐Se@DOX was significantly uptaken in HeLa cells with overexpressed folate receptors through the mesh protein‐mediated endocytosis pathway, compared to lung cancer A549 cells with deficient folate receptors. FA‐Se@DOX significantly inhibited HeLa cells’ proliferation, while causing cell apoptosis compared with DOX or Se@DOX at the same dose. Moreover, FA‐Se@DOX was able to specifically accumulate at the tumor site, resulting in significant anti‐tumor efficacy in vivo compared with any other treatment group. Mechanically, as shown in the results of p53, caspase‐3, and TUNEL assays, FA‐Se@DOX was demonstrated to induce cancer cell apoptosis obviously. Memon et al. [139] synthesized chitosan‐modified Se nanoparticles loaded with paclitaxel (PTX‐chit‐SeNPs) and demonstrated their role in promoting apoptosis in HeLa cells by upregulating Bax gene expression. Bidkar et al. [140] synthesized PTX‐loaded SeNPs and found their antiproliferative activity against HeLa cells. Mechanistically, treatment with PTX‐SeNPs resulted in dose‐dependent G2/M phase arrest, ultimately triggering cellular apoptosis and disrupting mitochondrial membrane potential due to ROS induction, causing caspase activation.

Nasrolahi et al. [141] synthesized a cyclic peptide composed of five tryptophans, four arginines, and one cysteine [W5R4C], and generated cyclic peptide‐capped Se nanoparticles ([W5R4C]‐SeNPs) in situ. The antiproliferative results revealed that compared to anticancer drugs treatment alone, all anticancer drugs, including clofarabine, DOX, etoposide, gemcitabine, epirubicin, fludarabine, camptothecin, dasatinib, irinotecan, and PTX, combined with [W5R4C]–SeNPs, significantly inhibited cell proliferation in SKOV3 cells due to the improvement of cellular uptake of the drugs by [W5R4C]‐SeNPs. Further, degradation of the peptide‐capped SeNPs complex can produce toxic Se aggregates in cancer cells that kill them synergistically (Figure 4C).

### 5.4 Combine With Photothermal Therapy

In photothermal therapy, light (typically near infrared, NIR) is used to increase tissue temperature and achieve localized photocoagulation, thus eliminating cancer cells with spatiotemporal precision [146, 147]. In contrast to conventional therapeutic methods, the precise targeting characteristic of photothermal therapy provides an attractive alternative for treating gynecological cancers, and it is particularly beneficial for preserving ovarian function in young female patients [148]. In our previous study [142], gold‐Se coordination bonds were used to fabricate immunogenic core–shell Au@Se nanoparticles. This Se‐incorporated gold nanosystem (Au@Se NPs) demonstrated enhanced photothermal stability and improved conversion efficiency, thereby facilitating the decomposition and conversion of Se, resulting in higher thermotherapy after being combined with PTT to enhance the apoptosis of cervical cancer cells HeLa. Under an 808 nm NIR laser, Au@Se NPs severely damaged tumor cells, leading to extensive apoptosis in U14 cells tumor‐bearing mouse models. Mechanistically, Au@Se NPs combined with NIR laser‐induced cleavage of caspase‐3/‐7/‐9 and PARP in HeLa cells, demonstrating synergistic antitumor effects mediated through caspase‐dependent apoptotic pathways. It was also demonstrated that Au@Se NPs + NIR laser irradiation killed cancer cells more effectively than those treated alone, with ROS levels much higher than those in any other group. In vivo studies were conducted using U14 tumor‐transplanted mouse models to explore the further functions of the nanosystem combined with laser therapy. Interestingly, compared to other groups, Au@Se NPs combined with NIR laser treatment markedly facilitated the maturation of DCs and significantly enhanced the infiltration of cytotoxic T lymphocytes (CTLs) compared with other treatment groups. In addition, the percentage of CD4^+^ T cells was also upregulated, which was a fivefold increase compared to the saline treatment group and a twofold increase compared to other treatment groups. Upon reaching a tumor volume of approximately 0.1 cm^3^, mice received subcutaneous injections of U14 cells on the contralateral side to establish a distant tumor model. CD8^+^ T and CD4^+^ T cells exhibited significant infiltration into distant tumors, while the proportion of M2 macrophages was markedly reduced following intratumoral administration of Au@Se NPs under NIR laser irradiation. Li et al. [143] synthesized a drug delivery platform based on bismuth selenide (Bi_2_Se_3_) NPs coated with polydopamine (PDA)/human serum albumin (HSA)/DOX, possessing strong NIR absorbance ability, high stability, and efficient photothermal conversion capabilities suitable for PTT applications. Within 10 min, the temperature of Bi_2_Se_3_@PDA/DOX/HSA suspension increased significantly to 50.0°C under 808 nm NIR laser irradiation with 1.2 W/cm^2^. After 5 min of NIR laser irradiation with Bi_2_Se_3_@PDA/DOX/HSA NPs, the combination treatment significantly inhibited HeLa cell viability. In the irradiated spot, most cells were destroyed, but those outside survived. After a 10‐minute NIR laser irradiation, cancer cells were destroyed completely. In vivo, compared to any single monotherapy alone, the Bi_2_Se_3_@PDA/DOX/HSA synergy with the NIR group effectively inhibited the growth of subcutaneously transplanted tumors established by HeLa cells. Yuwen et al. [144] prepared ultra‐small (2–3 nm) MoSe_2_‐nanometer dots (NDs). It was found that MoSe_2_ NDs had both a high extinction coefficient and a high photothermal conversion efficiency of NIR light during material characterization experiments. In comparison with the most studied plasmonic PTT agents, MoSe_2_ was a semiconductor material with excellent photothermal conversion properties, which proved its superior performance as a PTT reagent. What's more, water dispersion temperature remained stable during the NIR laser irradiation process, proving the photostability of MoSe_2_ NDs water dispersion of MoSe_2_ NDs. Increasing concentrations of MoSe_2_ NDs continuously decreased the HeLa cells’ viability under a 785 nm NIR laser for 10 min. Of note, MoSe_2_ NDs reduced the HeLa cells’ viability to less than 8% at a relatively low concentration of 40 mg/mL, proving their potential as excellent nano‐agents for photothermal therapy (Figure 4D).

### 5.5 Combine With Chemodynamic Therapy

Chemodynamic therapy (CDT), as an emerging tumor treatment modality, operates through the catalytic conversion of endogenous chemical substrates within tumors via Fenton or Fenton‐like reactions, which induce the generation of·OH from H_2_O_2_ to kill cancer cells [149, 150, 151]. For instance, Zheng et al. [145] designed a multifunctional biomimetic nanozyme (SSMA/DOX). Under acidic tumor conditions, the SSMA/DOX nanosystem reactively degraded in cancer cells to release Se, DOX, Au, and Mn. Therefore, it catalyzed the conversion of endogenous H_2_O_2_ to ·OH for CDT. Of note, by accumulating ROS in cervical cancer HeLa cells, Se significantly enhanced the chemotherapy of DOX based on CDT, exerting a significant inhibitory effect on cancers. In vivo, the tumors treated with the SSMA/DOX nanocomposites completely disappeared after 14 days in HeLa tumor‐bearing mice. Thus, Se‐based nanoparticles‐mediated CDT for antitumor application has a promising therapeutic prospect to enhance tumor therapy (Figure 4E).

## 6 Se‐Based Nanoparticles for Gynecological Cancer Early Diagnosis and Imaging

Beyond their applications in anti‐tumor therapy, Se‐based nanoparticles also serve as effective contrast agents and probes for molecular diagnosis and imaging of gynecological cancers, thereby enabling early cancer screening and detection (Table 4).

**TABLE 4:** Summary of Se‐based nanoparticles for cancer early diagnosis and imaging.

Applications	Nanomaterials	Cancer Types	Refs.
Early molecular diagnosis	AuNP@SiO_2_	ovarian cancer	[152]
	Cd‐Se/ZnS	ovarian cancer	[153]
Fluorescence imaging	Cd‐Se	cervical cancer	[154]
	SeDSA	cervical cancer	[155]
	Cd‐Se/ZnS	ovarian cancer	[156]
CT imaging	Bi_2_Se_3_@PDA/DOX/HSA NPs	cervical cancer	[143]
MRI imaging	Se@SiO_2_‐Mn@Au	cervical cancer	[145]

### 6.1 Application in Early Molecular Diagnosis

**FIGURE 5:**
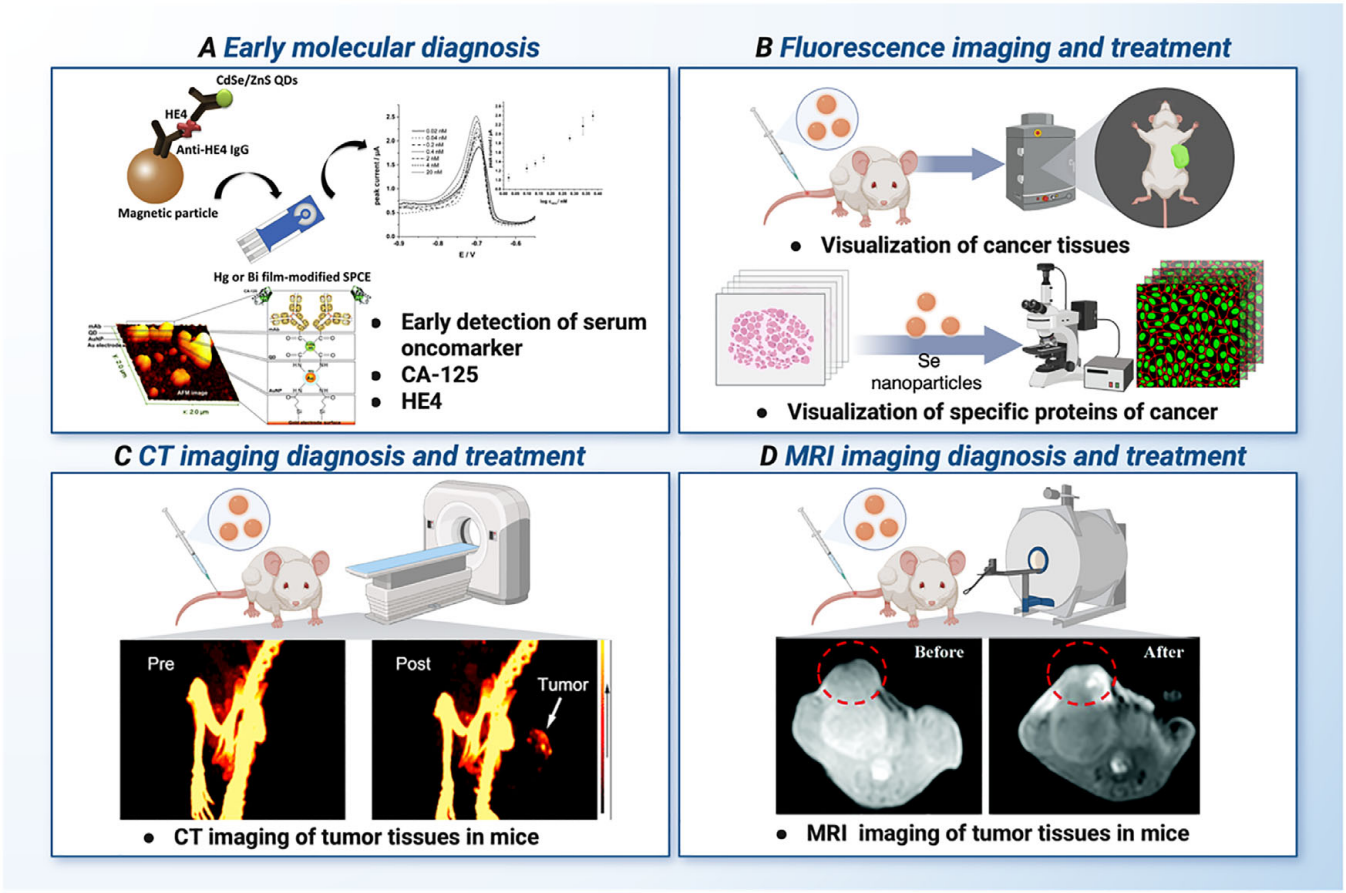
Applications of Se‐based nanoparticles for gynecological cancer early diagnosis and imaging. (A) Application in early molecular diagnosis. (B) Application in fluorescence imaging and treatment. (C) Application in CT imaging diagnosis and treatment (D) Application in MRI imaging diagnosis and treatment. Part A were reproduced with permission. [152], copyright 2018, Royal Society of Chemistry (RSC), and [153], Copyright 2018, Elsevier. Part C was reproduced with permission. [143], Copyright 2015, American Chemical Society. Part D was reproduced with permission. [145], Copyright 2022, Royal Society of Chemistry. (Others were created with BioRender.com).

Early detection of ovarian cancer is crucial to effective treatment. Currently, the most effective diagnostic approach for ovarian cancer remains the combined use of cancer antigen 125 (CA125) and Human Epididymis Protein4 (HE4) [157, 158]. Johari‐Ahar et al. [152] reported a new nanostructured immunosensor that can detect serum oncomarker CA125 at an early stage. Biosensors are technical devices that convert biological functions (e.g., interactions between biomolecules) into electrical signals that can be detected. In this study, a gold electrode modified with mercaptopropionic acid (MPA) was sequentially conjugated consecutively with silica‐coated gold nanoparticles (AuNP@SiO_2_), Cd‐Se QDs, and anti‐CA125 monoclonal antibody (mAb) to enhance the detection sensitivity, enabling the quantification of trace levels of CA125. Using this approach, CA125 was specifically and sensitively detected at a limit of detection (LOD) value of 0.0016 U/mL. The approval of HE4 as an ovarian cancer biomarker holds substantial clinical significance, as it demonstrates the capacity to differentiate malignant from benign conditions and reflects the differentiation stage of the neoplastic disease. For the purpose of sensitive HE4 detection and quantification in human serum, Cadkova et al. [153] developed an enzyme‐free electrochemical immunosensor. For HE4 capture, core/shell Cd‐Se/ZnS QDs were conjugated with anti‐HE4 IgG antibodies to facilitate sandwich‐type immunosensing. Specifically, electrochemical detection of HE4‐anti‐HE4 IgG^CdSe/ZnS^ immunocomplex was performed by recording the current response of Cd^2+^ ions, which were released from dissolved QDs. This approach enabled the rapid and sensitive quantification of HE4 biomarker in the early stage of ovarian cancer. (Figure 5A)

### 6.2 Application in Fluorescence Imaging and Treatment

The fluorescence imaging technique is used to visualize invisible tumor cells or proteins. It is possible to diagnose cancer cells accurately using fluorescent antibodies or fluorescent labeling reagents by analyzing cellular morphology, structure, and biological activity [69, 159]. In contrast to conventional organic contrast agents, semiconductor nanocrystals, including Se‐based ones, possess distinctive optical characteristics that are essential for biological applications requiring ultrahigh sensitivity. The properties of QDs have emerged as highly attractive molecular probes for cellular labeling in biological research owing to their exceptional optical characteristics. Vibin et al. [154] explored the cellular uptake and subcellular distribution of silica‐coated Cd‐Se QDs in cervical cancer cells and found that these Cd‐Se QDs were efficiently taken up by the cells and sequestered within intracellular vesicles. Subsequently, they emitted strong fluorescence from both the cytoplasm and the nuclei. Han et al. [155] successfully synthesized fluorescent diselenide 9, 10‐styrene‐artemisia (DSA) derivatives (SeDSA) containing diselenides with aggregation‐induced emission (AIE) properties, and SeDSA NPs were prepared by the nanoprecipitate method. Due to the AIE characteristics of SeDSA and the redox response characteristics of the diselenide bond, they could be easily uptaken by HeLa cells and showed intense orange fluorescence emission, suggesting potential applications in cell imaging.

According to Generalova et al. [156], highly fluorescent polymer particles were fabricated by encapsulating Cd‐Se/ZnS QDs within polyacrolein and poly(acrolein‐co‐styrene) polymeric matrices. QD‐embedded polymer particles demonstrated specific targeting of HER2/neu cancer markers on SKOV3 cells following 4D5 scFv antibody conjugation, enabling subsequent fluorescence microscopy imaging of ovarian cancer cells (Figure 5B).

### 6.3 Application in CT Imaging Diagnosis and Treatment

As a representative topological insulator, bismuth selenide (Bi_2_Se_3_) possesses a Dirac cone structure and a bulk band gap of 0.3 eV, characteristics that have significant relevance for computed tomography (CT) imaging applications [143]. Based on this character, Li et al. [143] developed a drug delivery nanosystem using Bi_2_Se_3_ nanoparticles coated by PDA/HSA/DOX. Since the Bi element has a high X‐ray attenuation coefficient, the incremental CT signal value intensity increased with increasing NPs concentration. These NPs had an X‐ray absorption coefficient of 7.53 HU mmol/L, which was significantly higher than that of the commercial contrast agent (iohexol, 3.93 Hounsfield unit mmol/L). A micro‐CT scanner was employed to visualize BALB/c mice bearing HeLa tumors that had received intratumoral injections of the aforementioned nanoparticles. The 3D CT image scans revealed significantly enhanced contrast at the tumor location, accompanied by substantially elevated CT attenuation values. In addition, the CT images were also taken from tumor‐bearing mice treated by intravenous. As the NPs passively accumulated into the tumor through the EPR effect over time, resulting in a progressive increase in mean Hounsfield unit values, reaching 126.8 HU at 18 hours post‐injection (Figure 5C).

### 6.4 Application in MRI Imaging and Treatment

Zheng et al. [145] successfully synthesized a TME‐responsive degradable theranostic agent based on Se@SiO_2_‐Mn@Au nanocomposites (SSMA NCs). Further study demonstrated the magnetic resonance imaging capabilities of SSMA NCs in HeLa tumor‐bearing mice. Compared to injection before, an obvious T1‐weighted magnetic resonance imaging (MRI) signal was observed within the tumor region of mice bearing tumors after injection of SSMA NCs, indicating its excellent MRI image capability for use in vivo therapy (Figure 5D).

## 7 Conclusion and Perspectives

Overall, as an important trace element, Se deficiency is considered closely associated with the pathogenesis and progression of gynecological cancers. Multiple prospective studies have shown its role in reducing the adverse effects of radiotherapy and chemotherapy. Se supplementation during chemoradiotherapy may be an effective therapeutic strategy against the adverse effects of chemotherapy or radiotherapy. In addition, Se is a semiconductor with favorable electrical conductivity, notable photovoltaic and photoconductive properties, thermoelectric responsiveness, and remarkable oxidative properties. Functionalized Se‐based nanoparticles show excellent biodegradability, biocompatibility, and lower toxicity. Further, they could target cancer sites by active or passive targeting, or precisely release active pharmaceutical ingredients by tumor microenvironment‐response targeting. Therefore, functionalized Se‐based nanoparticles exert anti‐tumor effects through direct killing, radiosensitization, chemotherapy drug delivery, chemotherapeutic sensitization, combined with photothermal therapy and chemodynamic therapy. Additionally, functionalized Se nanoparticles can serve as a versatile platform for the diagnosis and therapy of gynecological cancers, including early molecular diagnosis, fluorescence imaging, CT, and MRI‐guided diagnosis and concurrent treatment.

Although Se and Se‐based nanoparticles are highly promising in tumor therapy, further advancements and studies are necessary. Further research is urgently needed in the following aspects. Various studies have come to different conclusions regarding the risk of gynecological cancers associated with Se intake. As a matter of fact, Se levels in human samples and Se intake have a high degree of global variability due to factors such as dietary habits, soil and food Se content, ethnicity, gender, age, metabolism, occupational exposure, coal exposure, and smoking exposure. Heavy metals (such as arsenic, cadmium, and mercury) and dietary factors (such as methionine) may modify Se's effects, potentially confounding its cancer‐related effects [160]. Therefore, these results must be interpreted with great caution, and further comprehensive research will be appropriate to conduct.

**FIGURE 6:**
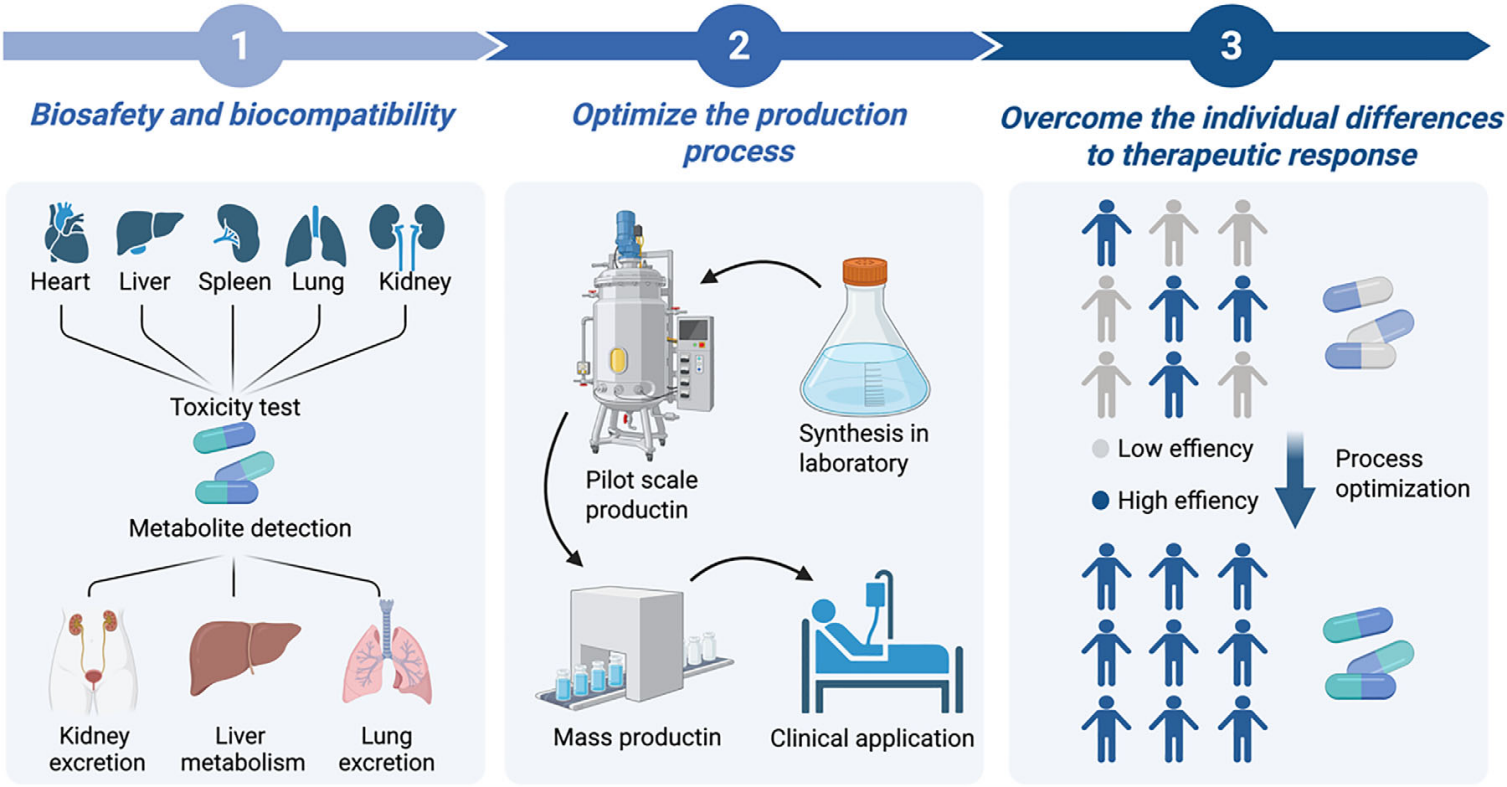
The future development directions of Se‐based nanoparticles in early diagnosis and effective treatment of gynecological malignant tumors. (created with BioRender.com).

At present, researches on Se‐based nanoparticles are limited in laboratories, and how to verify their anti‐cancer activity in clinical remains a difficult problem. (1) Nanomaterials' toxicity and safety hinder their practical application in clinics. NPs must ideally be biodegradable while avoiding biological agglomeration and adverse side effects. Multiple studies have noted the metabolic process of Se element after Se‐based nanoparticles treatment in vitro and in vivo, that the Se element has good biosafety and biocompatibility due to its degradability. However, the metabolic processes of other elements in the Se nanocomposites still need to be studied. (2) The synthesis and purification of engineered nanoparticles require complicated processes, making it difficult to generate products of consistent quality on a large scale, which further limits their effectiveness in clinical trials. Excitingly, He et al. [161] successfully scaled the synthesis of Se nanoparticles (SeNPs) to pilot and 250‐liter industrial levels, demonstrating that lentinan's confined coordination structure plays a crucial regulatory role in controlling SeNPs nucleation, growth processes, and material stability. This research offers a scientific foundation for future development and production of nanomedicines. 3) Due to the heterogeneity of tumor microenvironments and the diversity of cancer, different parts of the cancer or different individuals respond differently to the same medication. To achieve a satisfactory therapeutic response, NPs must take into account individual differences. After overcoming the above problems, it is believed that Se‐based nanoparticles can be applied in the clinical treatment of gynecological malignant cancers in the future. It will provide broad prospects for improving the early diagnosis and effective treatment of gynecological malignant cancers (Figure 6).

## Author Contributions

Hejing Liu, Yujia Zhou, Zihan Zhang, Liqing Miao, Rong Ma and Luyi Ruan searched the literature. Hejing Liu and Yujia Zhou made the figures. Yujia Zhou, Boxin Zhang and Huihui Ji made the tables. Hejing Liu wrote the manuscript. Xueqiong Zhu and Tianfeng Chen revised the manuscript. All authors read and approved the final manuscript.

## Funding

The review received funding from the National Health Commission Scientific Research Fund—Zhejiang Provincial Health Major Science and Technology Plan project (WKJ‐ZJ‐2510).

## Ethics Statement

The authors have nothing to report.

## Consent

The authors have nothing to report.

## Conflicts of Interest

The authors declare no conflicts of interest.

## Data Availability

Data sharing not applicable to this article as no datasets were generated or analyzed during the current study.
